# New insights into the distribution, protein abundance and subcellular localisation of the endogenous peroxisomal biogenesis proteins PEX3 and PEX19 in different organs and cell types of the adult mouse

**DOI:** 10.1371/journal.pone.0183150

**Published:** 2017-08-17

**Authors:** Claudia Colasante, Jiangping Chen, Barbara Ahlemeyer, Rocio Bonilla-Martinez, Srikanth Karnati, Eveline Baumgart-Vogt

**Affiliations:** Institute for Anatomy and Cell Biology, Division of Medical Cell Biology, Justus Liebig University of Giessen, Giessen, Germany; Louisiana State University Health Sciences Center, UNITED STATES

## Abstract

Peroxisomes are ubiquitous organelles mainly involved in ROS and lipid metabolism. Their abundance, protein composition and metabolic function vary depending on the cell type and adjust to different intracellular and environmental factors such as oxidative stress or nutrition. The biogenesis and proliferation of these important organelles are regulated by proteins belonging to the peroxin (PEX) family. PEX3, an integral peroxisomal membrane protein, and the cytosolic shuttling receptor PEX19 are thought to be responsible for the early steps of peroxisome biogenesis and assembly of their matrix protein import machinery. Recently, both peroxins were suggested to be also involved in the autophagy of peroxisomes (pexophagy). Despite the fact that distribution and intracellular abundance of these proteins might regulate the turnover of the peroxisomal compartment in a cell type-specific manner, a comprehensive analysis of the *endogenous* PEX3 and PEX19 distribution in different organs is still missing. In this study, we have therefore generated antibodies against *endogenous* mouse PEX3 and PEX19 and analysed their abundance and subcellular localisation in various mouse organs, tissues and cell types and compared it to the one of three commonly used peroxisomal markers (PEX14, ABCD3 and catalase). Our results revealed that the abundance of PEX3, PEX19, PEX14, ABCD3 and catalase strongly varies in the analysed organs and cell types, suggesting that peroxisome abundance, biogenesis and matrix protein import are independently regulated. We further found that in some organs, such as heart and skeletal muscle, the majority of the shuttling receptor PEX19 is bound to the peroxisomal membrane and that a strong variability exists in the cell type-specific ratio of cytosol- and peroxisome-associated PEX19. In conclusion, our results indicate that peroxisomes in various cell types are heterogeneous with regards to their matrix, membrane and biogenesis proteins.

## Introduction

Peroxisomes are single membrane-bound organelles that can either be formed *de novo* or multiply by fission [1]. The proliferation of peroxisomes, the assembly of their membrane and the import of peroxisomal matrix enzymes into the organelle are regulated by proteins belonging to the family of peroxins (PEX-proteins) [2,3]. In yeast, mice and humans, more than 32 different genes coding for peroxins have been identified, which are either integral part of the peroxisomal membrane or soluble cytosolic receptors [2,3] (http://www.ncbi.nlm.nih.gov/protein). Though many key players of the peroxisomal biogenesis have been already discovered 25 years ago, the question on how they functionally interact and how peroxisomes are formed *de novo*, how their membrane is generated and how they divide by fission is still not fully clarified [1]. Two peroxins were identified and intensively studied that interact and closely cooperate during the initial steps of yeast [4–7] and mammalian [8–10] peroxisome biogenesis, namely PEX3 and PEX19 [11]. Cells in which these peroxins had been depleted, lacked peroxisomes and complementation studies showed that the re-introduction of the respective gene into the knock-out background could restore *de novo* peroxisome biosynthesis [6,12,13]. The role for PEX3 and PEX19 in the formation of peroxisomes is the insertion of peroxisomal membrane proteins (PMPs) into the membrane of the nascent organelle [3,1]. In the initial steps of peroxisome formation, PEX19 binds PMPs in the cytosol through a peroxisomal membrane-targeting signal (mPTS) consisting of a PMP-binding domain and a membrane-anchoring domain [14–17]. PEX19 could also function as a chaperone, aiding the correct folding of PMPs [18,19]. The latest theory on how peroxisomes form *de novo* in yeast suggests that PEX3 might be autonomously integrated into the membrane of the ER from which PEX3-loaded pre-peroxisomal vesicles arise [1,20,21–24]. A more recent publication proposes that in mammalian cells *de novo* peroxisomal biogenesis begins with the budding of PEX3-loaded pre-peroxisomal vesicles from the mitochondrion, followed by their maturation to peroxisomal vesicles in the ER [25]. The exact mechanism is, however, not fully understood and still matter of debate [26]. PEX19 targets the bound PMPs to pre-peroxisomal vesicles and inserts them into the peroxisomal membrane by docking to PEX3 [1,4,27]. These initial steps of peroxisome biogenesis lead to the integration of peroxisomal substrate transporters into the membrane and to the assembly of the machinery necessary for the import of matrix proteins. This import complex consists of other proteins of the peroxin family (e.g. PEX14) and initiates the loading of the newly formed peroxisomes with soluble matrix enzymes [3,28].

Enzymes that are imported into the peroxisomal matrix take part in different metabolic pathways such as the scavenging of reactive oxygen species (ROS), β-oxidation of fatty acids or the synthesis of glycerolipids and cholesterol precursors [29]. Despite the fact that peroxisomes of different organs share certain common features, the organelle’s proteome is fine-tuned depending on the metabolic demand of the organ or cell type [30–33]. For example: peroxisomes of the liver and of the proximal tubules of the nephron, the organs in which peroxisomes were first described, contain high amounts of catalase. For this reason catalase has been used as marker enzyme in many studies performed on peroxisomes in the past years. The amount of peroxisomal catalase, however, can vary between cell types [34–39] and low catalase content does not necessarily correlate with a less developed peroxisomal compartment [39]. Differential protein expression depending on the cell´s developmental and nutritional state was also found for the peroxisomal membrane transporter ABCD3 [34,39–42]. Peroxins play a key role in regulating the turnover of peroxisomes and are therefore likely to be differentially expressed in different organs depending on metabolic requirements. Moreover, peroxisomes in different cell types vary in their morphological appearance from round to rod-shaped to tubular and network-like [43], changing the relative matrix to membrane volume ratio, which might also affect the quantity of peroxins associated with the peroxisomal membrane [44].

Although intensive investigations have been made to study the function of PEX3 and PEX19 in model organisms such as the yeast *Saccharomyces cerevisiae* little is known about their organ-specific distribution and function in mammalian cells and organs [1]. Furthermore, besides cell culture studies, endogenous PEX3 and PEX19 have not yet been investigated in mammalian organs. Determining presence and amount of these proteins in different organs and specific cell types could provide an indication on early biogenesis steps, proliferation state and turnover rate of their peroxisomes. Since PEX3 and PEX19 are involved in peroxisome biogenesis, we expected their highest expression in organs whose cells either contain a large number of peroxisomes such as liver and kidney or with a high rate of peroxisome remodelling and renewal. For this reason, organs and cell types containing high amounts of PEX3 and PEX19 could be more frequently negatively affected by defects of the peroxisomal biogenesis such as occur in diseases of the Zellweger syndrome spectrum and neonatal adrenoleukodystrophy [45]. Patients affected by these devastating hereditary disorders do not possess functional peroxisomes [46], which results in systemic metabolic dysfunctions that lead to multiple organ defects and early death of the affected children [45]. Indeed, mutations in the *PEX3* [47,48] and the *PEX19* [49] genes lead to the most severe form of the peroxisomal biogenesis disorders, the Zellweger syndrome.

In the past years, we have tested out several antibodies against PEX3 and PEX19 that all did not give satisfactory results for morphological stainings of the endogenous proteins. Therefore, we have generated anti-PEX3 and anti-PEX19 antibodies specific and sensitive enough to analyse organ- and cell type-specific distribution and abundance of these peroxins *in situ*. Our results showed that PEX3 and PEX19 protein abundance and mRNA expression were differentially regulated in different organs and cell types. We also found that organs in which common peroxisomal marker proteins were highly expressed or that contained a large number of peroxisomes did not necessarily display high levels of PEX3 and PEX19 and that the ratio between peroxisomal membrane-associated and cytosol-located PEX19 conspicuously varies between different organs and cell types. In conclusion, organ- and cell type-specific adaptations of the peroxisomal protein composition do not only concern their metabolic but also their peroxisome biogenesis proteins (peroxins), which do not seem to be regulated in a strictly orchestrated manner but rather independently from each other.

## Materials and methods

### Animals

For the isolation of the organs, 3 male 19 weeks-old C57BL/6J mice were obtained from the central animal facility (Zentrales Tierlabor—ZTL) of the Justus Liebig University of Giessen. Animals were housed under standard conditions (12 h light and 12 h dark cycle) with free access to food and water. Additionally, 2 pregnant female C57BL/6J mice were sacrificed to obtain 20 E14 embryos used for the isolation of mouse embryonic fibroblasts (MEFs). All experiments with laboratory mice were approved by the German Government Commission of Animal Care (V54-19c 20/15c GI20/23; University internal classification: JLU-Nr.: 471_M, Project ID: 1016 Peroxisomen).

### Generation of serum polyclonal antibodies directed against PEX3 and PEX19

The open reading frame coding for amino acids 35 to 372 of PEX3 (NM_019961.3) was amplified from pCMV-Sport 6 containing the *Pex3* cDNA (I.M.A.G.E. clone IRAKp961A2345Q, now IRAVp968C0184D) using the forward primer (gcgCATATGcaagaaagagaagctgcagaatacattg) and the reverse primer (cgcGGATCCtcatttctccagttgttggggggtactaaac). The open reading frame coding for full-length PEX19 (NM_023041.3) was amplified from pCMV-Sport 6 containing the *Pex19* cDNA (I.M.A.G.E. clone IRAKp961I1541Q, now IRAVp968E0238D) using the forward primer (gcgCATATGgcggctgctgaggaaggttg) and the reverse primer (cgcGGATCCctacatgatcagacactgttcg). Both PCR products were cloned into the bacterial expression vector pET16b (Invitrogen) using the restriction enzyme sites *Nde*I and *BamH*I included in the primer sequences (capital and underlined). This results in the addition of 10 N-terminal histidine residues to the protein. The *Escherichia coli* strain BL21 (DE3) was transformed with the obtained plasmids pET16b_PEX3^35-372^ (named pEBV13) and pET16b_PEX19 (named pEBV15). The expression of PEX3 and PEX19 was induced in 500 ml transformed bacterial cultures grown in Terrific Broth medium containing 10 mM malate and 10 mM pyruvate at an OD_600_ of 0.4 at 37°C using 1 mM isopropyl-β-D-thiogalactopyranosid (IPTG). The bacteria expressing PEX19-10xHis and PEX3^35-372^-10xHis were harvested 180 min post-induction by centrifugation at 5,000 x *g* for 15 min. The bacteria were resuspended in Dynabeads Binding/Washing buffer (50 mM Na-phosphate, 300 mM NaCl, 0.01% (v/v) Tween 20, pH 8.0) supplemented with 10% protease inhibitor mix, 100 mg/ml lysozyme and 300 U DNase I. The cells were incubated on ice for 20 min and lysed by sonication (10 times 10 s at 50% output). After removal of the cell debris by centrifugation at 5,000 x *g* for 15 min, the supernatant was incubated for 30 min at 4°C with 100 μl Dynabeads (Invitrogen) pre-equilibrated in Dynabeads Binding/Washing buffer. After the incubation, the supernatant was removed and the Dynabeads were washed 5 times with Dynabeads Binding/Washing buffer. PEX3^35-372^-10xHis and PEX19-10xHis proteins were eluted from the column using 150 mM imidazole in Dynabeads Binding/Washing buffer. Then, 1 mg of the PEX3^35-372^-10xHis and PEX19-10xHis proteins were used to immunize two rabbits and two rats, respectively. The proteins were injected in 3 boosts at day 20, 30 and 40 after the first immunization. The serum was obtained 135 days after starting the immunization.

### Cloning of PEX3 and PEX19 into the pCI-Neo vector for mammalian over-expression

The open reading frames coding for PEX3 (NM_019961.3) and for PEX19 (NM_023041.3) were excised from pCMV-Sport6 (for I.M.A.G.E. clone number see above) using the restriction enzymes sites *Sal*I and *Not*I. The digested DNA fragments were then ligated into the mammalian expression vector pCI-Neo using the *Sal*I and *Not*I restriction enzyme sites located at its multiple cloning site generating the two expression vectors pCI-Neo_PEX3 (named pEBV74) and pCI-Neo_PEX19 (named pEBV47).

### Isolation, culturing and transfection of mouse embryonic fibroblasts

For the isolation of MEFs, we used mice on day 14 *post coitum*. The embryos were taken out, placed in a Petri dish and sacrificed by decapitation. The organs were removed and the remaining body was rinsed in phosphate-buffered saline (PBS) without Ca^2+^ and Mg^2+^. The rest of the body was digested with 0.05% trypsin/EDTA containing 100 U DNase I per embryo at 37°C for 15 min. Cells were dissociated by pipetting and transferred to MEF medium (high glucose DMEM supplemented with 10% foetal bovine serum, 100 U/ml penicillin/streptomycin, 1 mM sodium pyruvate and 2 mM L-glutamine) and cultivated at 37°C with 5% CO_2_. MEFs were transfected at 70% confluency 24 h after splitting and seeding into 6-well plates with 1 μg plasmid DNA using Lipofectamine 3000 (Life Technologies) according to manufacturer´s protocol. For immunofluorescence analysis cells were grown in MEF medium in 6-well plates on glass coverslips coated with 0.1% gelatin in PBS.

### Cell culture and transfection of Hepa 1–6 mouse hepatoma cells

Hepa 1–6 cells were cultivated in DMEM supplemented with 2 mM glutamine, 10% FBS and 100 U/ml penicillin/streptomycin at 37°C with 5% CO_2_. For immunofluorescence analysis, cells were grown on glass coverslips coated with 0.08% collagen in PBS. Hepa 1–6 cells were transfected at 50% confluency 24 h after splitting and seeding into 6-well plates with either 1 μg shRNA (Qiagen, Cat. 336311) using *Trans*IT-LT1 Reagent (Mirus) or with 50 μM siRNA (Qiagen, Cat. 3195727) using Lipofectamine 3000 (Life Technologies) according to the manufacturer´s protocol. After transfection, the cells were incubated for either 48 h and 72 h (PEX19 shRNA) or 24 h and 48 h (PEX3 siRNA) according to the time point at which the maximal protein downregulation was achieved and detected by Western blotting.

### Organ isolation

Adult male animals were sacrificed by cervical dislocation and perfused for 30 s anterogradely with PBS through the left ventricle to remove blood cells. The organs (liver, spleen, pancreas, heart, lung, jejunum, colon, kidney, testis, skeletal muscle and brain) were removed and cut into different parts. One part of the organs was either shock-frozen in either RNAzol (Sigma-Aldrich) or in 25 mM Tris-HCl, 1 mM EDTA, 1 mM DTT, 250 mM sucrose, pH 7.8 for RNA or protein isolation, respectively. The other part of the organs was placed in paraformaldehyde (PFA) for morphological studies.

### RNA isolation from mouse organs and RT-qPCR analysis

The total RNA from the different mouse organs (less than 50 mg tissue stored at -80°C in RNAzol after dissection) was extracted using RNAzol according to the manufacturer´s protocol and subsequently treated with DNase I to remove traces of DNA. Since brain contains a large amount of lipids (myelin), total RNA of brain samples was isolated from the frontal neocortices of 19 weeks-old mice using the RNeasy Lipid Tissue Mini Kit (Qiagen, Cat. 74804) according to the manufacturer´s protocol with an optimized phenol/guanidine based lysis for fatty organs. In brief, excised neocortices were harvested and shock-frozen in liquid nitrogen und then stored at -80°C. The frozen tissue was mechanically disrupted with scissors in 1 ml QIAzol Lysis Reagent. The lysate was incubated at 56°C (shaking at 300 rpm) for 1 h. Thereafter, 700 **μ**l chloroform were added under vigorous shaking and the mixture was allowed to stand for 2–3 min following centrifugation at 5,000 x *g* for 15 min at 4°C. The aqueous phase was mixed with an equal volume of 70% ethanol, the mixture was thoroughly mixed and then transferred to an RNeasy Mini spin column. After centrifugation at 12,000 x *g* for 15 s, the flow-through was discarded and the column was washed once with buffer RW1 and twice with buffer RPE. Total RNA was eluted from the column with RNase-free water. The exact amount and the purity (230/260 ratio >1.7) of all RNA preparations were analysed using the NanoDrop ND-2000 spectrometer (peqlab). The quality of the isolated RNA was assessed by formaldehyde denaturing gel electrophoresis. Only RNA samples displaying no degradation were used for first-strand cDNA synthesis. First-strand cDNA was synthesized from 2.0 **μ**g DNase I-treated total RNA using random primers, dNTPs and 50 U MultiScribe reverse transcriptase (Applied Biosystems) in a final volume of 20 **μ**l. For quantitative RT-PCR, we used the Maxima SYBR Green/Fluorescein qPCR Master Mix (Thermo Fisher Scientific), which was mixed 1:1 with the template cDNA, the forward and reverse primers and water. All samples were run in triplicates. The PCR reaction was performed in the IQ5 iCycler (BioRad Laboratories) using the following 3-step amplification protocol: 2 min at 95°C (denaturation), 42 cycles of 15 s at 95°C (denaturation), 30 s at 60°C or 65°C (annealing) and 30 s at 72°C (extension). All primer pairs (S1 Table) were verified for specificity (showing a single peak in the melting curve analysis) as well as for their amplification efficiency by 10-fold dilutions series. Calculations of the relative gene expression were done by the 2^-ΔΔct^ method [50] using three different reference genes namely the transcription-related gene TATA-box binding protein (*Tbp*), the structure-related gene ribosomal protein L13 (*Rpl13*) and the gene peptidyl prolyl isomerase (*Ppia*). To best compare the results from the different tissues, data are shown in relation to each individual reference gene as well as to the means of all 3 reference genes.

### Preparation of protein samples

Western blot analyses were performed using either lysates from MEFs, Hepa 1–6 cells and whole organs, or using peroxisome-enriched and peroxisome-depleted fractions derived from various organs and obtained by differential centrifugation. For whole cell lysates, Hepa 1–6 cells and MEFs were grown to confluency. Cells were harvested using 0.05% trypsin, centrifuged at 500 x *g* for 5 min, washed with 1 x PBS and collected by centrifugation at 500 x g for 5 min. The cell pellet was resuspended in 100 μl 50 mM Tris pH 8.0, 150 mM NaCl, 0.1% Triton X-100 supplemented with 10% protease inhibitor mix and incubated on ice for 30 min. Cells were then homogenized by 10 strokes in a Dounce homogenisator and were centrifuged at 500 x *g* for 5 min to remove cell debris. Complete cell disruption was checked under the microscope. For whole organ lysates, 50 mg of each dissected organ were resuspended in 2 ml of 25 mM Tris, 1 mM EDTA, 1 mM DTT, 250 mM sucrose, pH 7.8 and shredded using an Ika Ultra-Turrax. The obtained lysate was homogenized by 30 strokes in a Dounce homogenisator and then spun down at 500 x *g* for 5 min to remove cell debris. Complete cell disruption was checked under the microscope. To obtain the peroxisome-enriched fractions, 1.75 ml of whole organ lysate were first spun at 1,000 x *g* at 4°C for 10 min to remove the nuclei. The postnuclear supernatant was first centrifuged at 5,000 x *g* at 4°C for 10 min to remove large mitochondria and then centrifuged at 35,000 x *g* at 4°C for 30 min to obtain a peroxisome-enriched pellet and a peroxisome-depleted supernatant containing soluble and microsomal proteins. The pellet containing the peroxisomes was resuspended in 200 μl 20 mM Tris, 150 mM NaCl, 0.01% Triton X-100, pH 7.8. The protein concentration of all samples was determined using the Bradford assay from Bio-Rad Laboratories according to the manufacturer´s protocol. All fractions were immediately frozen and stored at -80°C until the Western blot analysis.

### Western blot analysis

The protein samples were separated by SDS-PAGE and transferred to polyvinylidene difluoride membranes (Invitrogen). Membranes were then blocked for 1 h in 5% fat-free milk (Roth) in 20 mM Tris, 150 mM NaCl, 0.1% Tween 20, pH 7.8 (TBS-Tween), followed by incubation with primary antibodies against the His-Tag, the peroxisomal biogenesis proteins PEX3, PEX19 and PEX14, the peroxisomal matrix enzyme catalase, the mitochondrial matrix enzyme superoxide dismutase 2 (SOD2) and the cytosolic protein glyceraldehyde 3-phosphate dehydrogenase (GAPDH) (S2 Table) for 1 h at room temperature. The membranes were washed and incubated with the secondary antibody for 1 h at room temperature. Detection was performed depending on the enzyme conjugated to the secondary antibody (S3 Table), either with the Immun-Star-AP detection kit (Bio-Rad Laboratories) for secondary antibodies conjugated to alkaline phosphatase or the ECL detection kit (Invitrogen) for secondary antibodies conjugated to horseradish peroxidase. Protein bands were detected by exposing the membranes to Kodak BioMax films. To assure that equal amounts of protein were present, all membranes were stained with Coommassie Brilliant Blue (CBB) (Simply Blue Stain, Invitrogen) according to the manufacturer´s protocol after the Western blot procedure was completed.

### Preparation of morphological samples, immunofluorescence staining and image acquisition

The dissected organ pieces obtained from 3 mice were immersion-fixed overnight in 4% PFA, 2% sucrose in PBS. The next morning, the fixed organs pieces were embedded in paraffin (Paraplast Plus). Two μm sections were cut with a rotation microtome and mounted on Superfrost Plus (+) slides. Deparaffinized and rehydrated sections were processed for antigen retrieval with 0.01% trypsin for 10 min at 37°C, followed by microwaving for 3 x 5 min at 900 W in 10 mM citrate buffer, pH 6.0. Blocking of non-specific protein binding sites was performed by incubation with 4% bovine serum albumin (BSA) in TBS-Tween. Sections were incubated with primary antibodies against the peroxisomal biogenesis proteins PEX3, PEX19 and PEX14, the peroxisomal matrix enzyme catalase or the peroxisomal membrane protein ABCD3 (S2 Table) overnight at room temperature with 1% BSA in TBS-Tween and then with the appropriate fluorochrome-conjugated secondary antibodies (S4 Table). Finally the sections were counterstained with the nuclear dye Hoechst 33342 (1 μg/ml). For tissue-specific adjustments of the staining procedure refer to S5 Table. For the immunofluorescence staining of MEFs or Hepa 1–6 cells the medium was removed and cells grown on coverslips were rinsed with PBS and fixed with 4% PFA, 2% sucrose in PBS for 20 min at room temperature. After washing the coverslips with PBS, the cells were permeabilized with 0.1% Triton X-100/PBS for 20 min and then blocked with PBS containing 1% BSA (PBSA) for 20 min. The cells on the coverslips were incubated 1 h at room temperature with the primary antibodies against the peroxisomal biogenesis proteins PEX3, PEX19 and PEX14 and the peroxisomal matrix enzyme catalase (S2 Table) diluted in PBSA followed by washing with PBS and 1 h incubation at room temperature with the secondary antibody diluted in PBSA (S4 Table). Finally, the cells on the coverslips were washed in PBS and the nuclei were counterstained with 1 μg/ml Hoechst 33342. All images were taken with the DC40 camera of the fluorescence microscope (Leica DM RD, Leica Microsystems) and processed using Photoshop CS5.

## Results

### Generation of serum polyclonal antibodies against the peroxins PEX3 and PEX19

**Fig 1:**
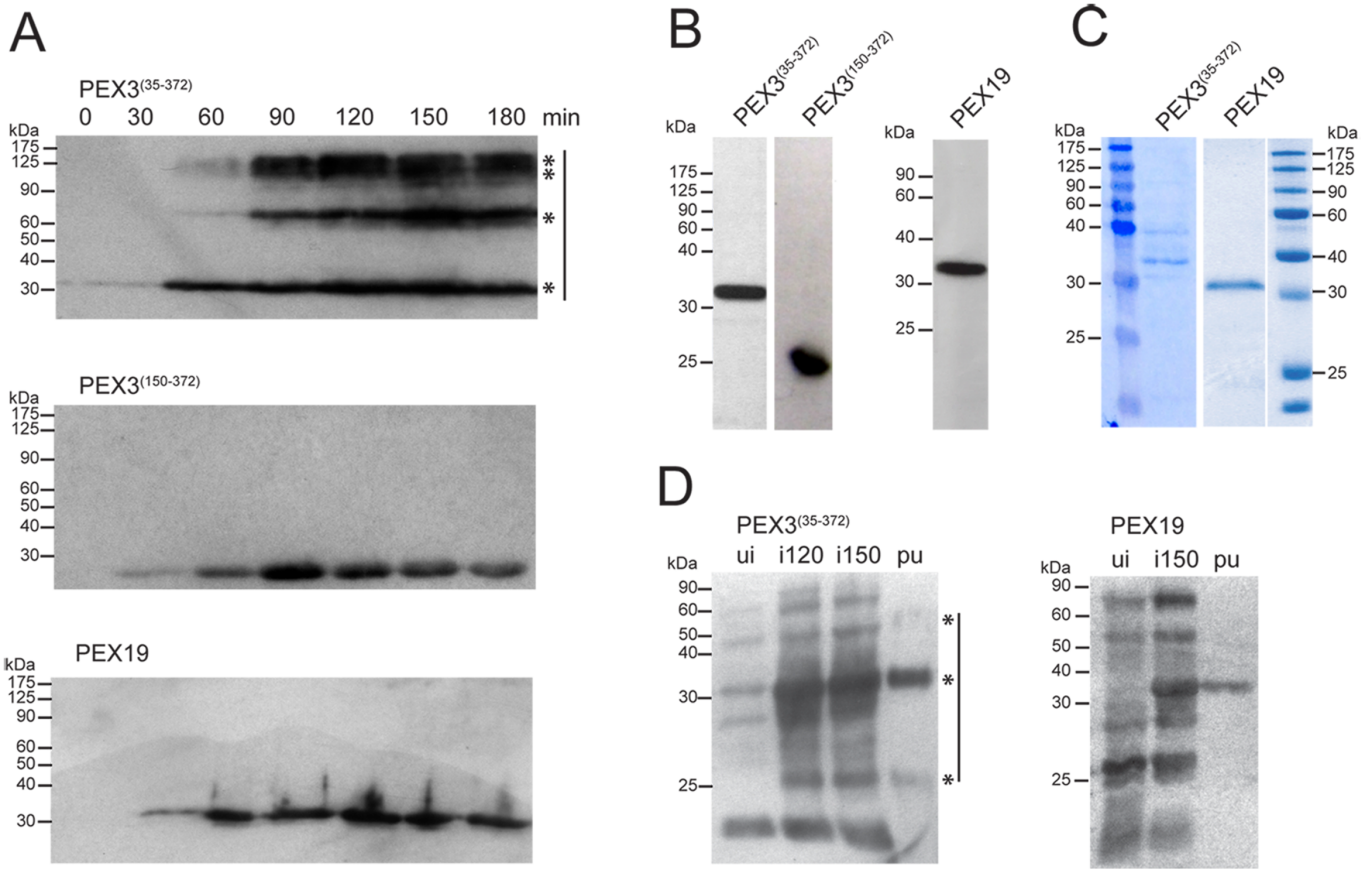
Recombinant PEX3^(35–372)^, PEX3^(150–372)^ and PEX19 were successfully expressed in BL21DE3 *E*. *coli* and purified for antibody generation by affinity chromatography. **A:** Time-dependent expression of PEX3^(35–372)^, PEX3^(150–372)^ and PEX19 in BL21DE3 *E*. *coli*. Bacterial samples were taken every 30 min post-induction with IPTG and lysates were analysed by SDS-PAGE and Western blotting with an antibody directed against the His-tag. Equal amounts of bacterial lysate calculated from the measured OD_600_ were loaded per lane. **B:** Isolated PEX3^(35–372)^, PEX3^(150–372)^ and PEX19. After purification of the expressed proteins using Talon magnetic Dynabeads, 10 μg of the protein samples were separated by SDS-PAGE and analysed by Western blotting using an antibody directed against the His-tag. **C:** CBB staining of the SDS gel depicting purified PEX3^(35–372)^ and PEX19. **D:** Expression of recombinant PEX3^(35–372)^ and PEX19 in BL21DE3 *E*. *coli* at different time points and detection of the purified proteins by Western blotting using the generated PEX3 and PEX19 antibodies. Ten μg of bacterial lysate were loaded prior (ui), as well as 120 (i120) and 150 (i150) min after the induction with IPTG, whereas 2 μg of purified eluted protein extracted from bacteria after 180 min of induction were loaded in lane pu. Asterisks (*) indicate bands that correspond to PEX3^(35–372)^ monomers and aggregates.

Most experiments that have investigated the function of PEX3 and PEX19 in mammalian systems have been performed using cell culture models and epitope-tagged overexpressed versions of these proteins [9,16,51,52]. The choice of using epitope-tagged versions of the two peroxins was partially due to unsatisfactory results obtained using commercially available antibodies. To investigate the subcellular and organ-specific distribution of endogenous PEX3 and PEX19, we first generated polyclonal antibodies from heterologously expressed proteins. For the generation of the antibody against PEX19, the whole open reading frame was expressed in *E*. *coli*. In contrast, since the expression of proteins that contain hydrophobic regions is notoriously difficult, for the membrane-associated PEX3, we opted for a truncated version (PEX3^35-372^), which lacked the putative peroxisome membrane anchor (amino acids 1–34). In *E*. *coli* the expression of short PEX3 versions was successfully carried-out for human PEX3 [19,53–55]. The time-dependent expression of murine PEX3^35-372^ (~32 kDa) and PEX19 (~35 kDa) was analysed by Western blotting using an antibody directed against the His-tag in whole bacterial lysates (Fig 1A). The abundance of both proteins increased over time after IPTG induction with a peak at 90–120 min. For PEX3^35-372^, 60 min after IPTG induction, increasing amounts of high molecular weight bands appeared (Fig 1A, upper panel), suggesting the formation of protein aggregates. High molecular weight bands have been previously reported in Western blot analyses of purified PEX3 [54,56]. It was suggested that PEX3 aggregates were formed due to its intrinsic capability to bind lipids and that they were induced by boiling the protein in SDS buffer [54,56].

Interestingly, high expression of a further truncated version of PEX3, PEX3^150-372^, in which the first 150 amino acids had been deleted, did not form high molecular weight bands (Fig 1A, central panel).

The purity of the isolated His-tagged PEX3^35-372^ and PEX19 proteins was assessed by Western blotting (Fig 1B) and CBB staining of the SDS gel (Fig 1C). The results show one prominent band of the expected molecular weight for both PEX3 and PEX19 in the Western blot analysis, while the CBB staining revealed additional bands for PEX3 but only one additional band for PEX19 (Fig 1B and 1C). The purified recombinant peroxins where used to immunize 2 rats (PEX3^35-372^) and 2 rabbits (PEX19).

The obtained antibodies were tested for their specificity using Western blot analysis of lysates from non IPTG-induced and IPTG-induced *E*. *coli* and of the purified protein. Both antibodies detect a time-dependent increase of a protein band of the expected molecular weight (32 kDa for PEX3; 33 kDa for PEX19) in the *E*. *coli* lysates (Fig 1D, lanes “ui” and “i120” and “i150”). The bands detected in lysates from non IPTG-induced *E*. *coli* (Fig 1D, lanes “ui”) were the result of “leakage” of the used expression system, while the additional bands detected in the bacterial lysates (Fig 1D, lanes “ui” and “i120” and “i150”), with similar amounts, irrespectively of the IPTG induction, were likely due to unspecific antibody binding. Both antibodies recognised a protein band of the expected molecular weight in the lane corresponding to the purified protein (Fig 1D, lanes “pu”). The antibody directed against PEX3 further recognized high molecular bands of ~55 and 60 kDa as well as one smaller of 25 kDa, which is probably a degradation product.

### The specificity of our self-generated antibodies against mouse PEX3 and PEX19 was ascertained by knockdown and overexpression experiments in MEFs and Hepa 1–6 cells

**Fig 2:**
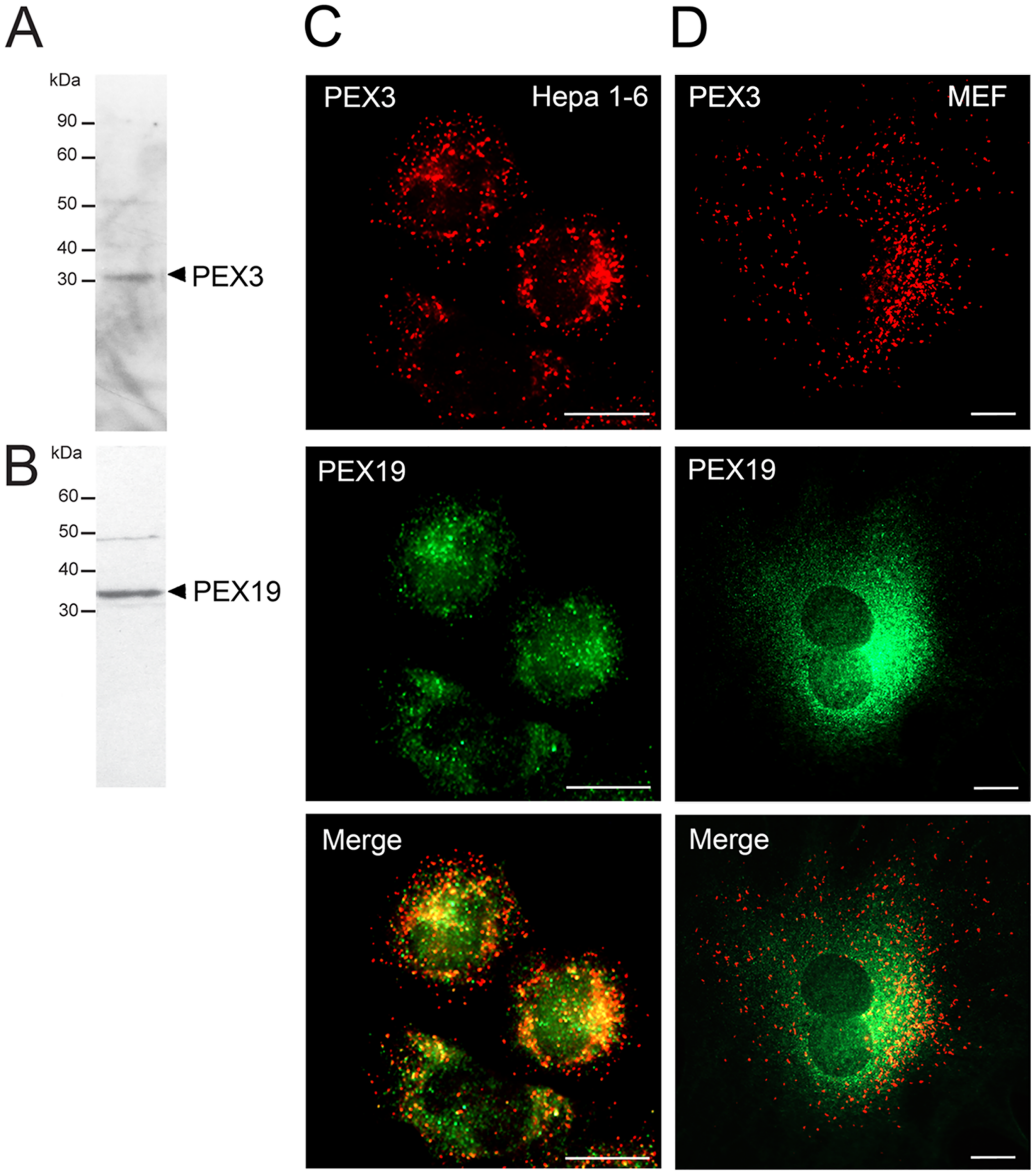
PEX3 and PEX19 are relatively low abundant and differently distributed in Hepa 1–6 cells and MEFs. **A and B:** Endogenous expression of PEX3 and PEX19 proteins in Hepa 1–6 cells. Hepa 1–6 cell lysates (20 μg protein) were loaded on an SDS gel and Western blotting was performed using the generated antibodies against PEX3 (A) and PEX19 (B). **C and D:** Double immunofluorescence stainings for PEX3 (red) and PEX19 (green) in Hepa 1–6 cells (C) and MEFs (D). **Scale bars** = 7.5 μm.

**Fig 3:**
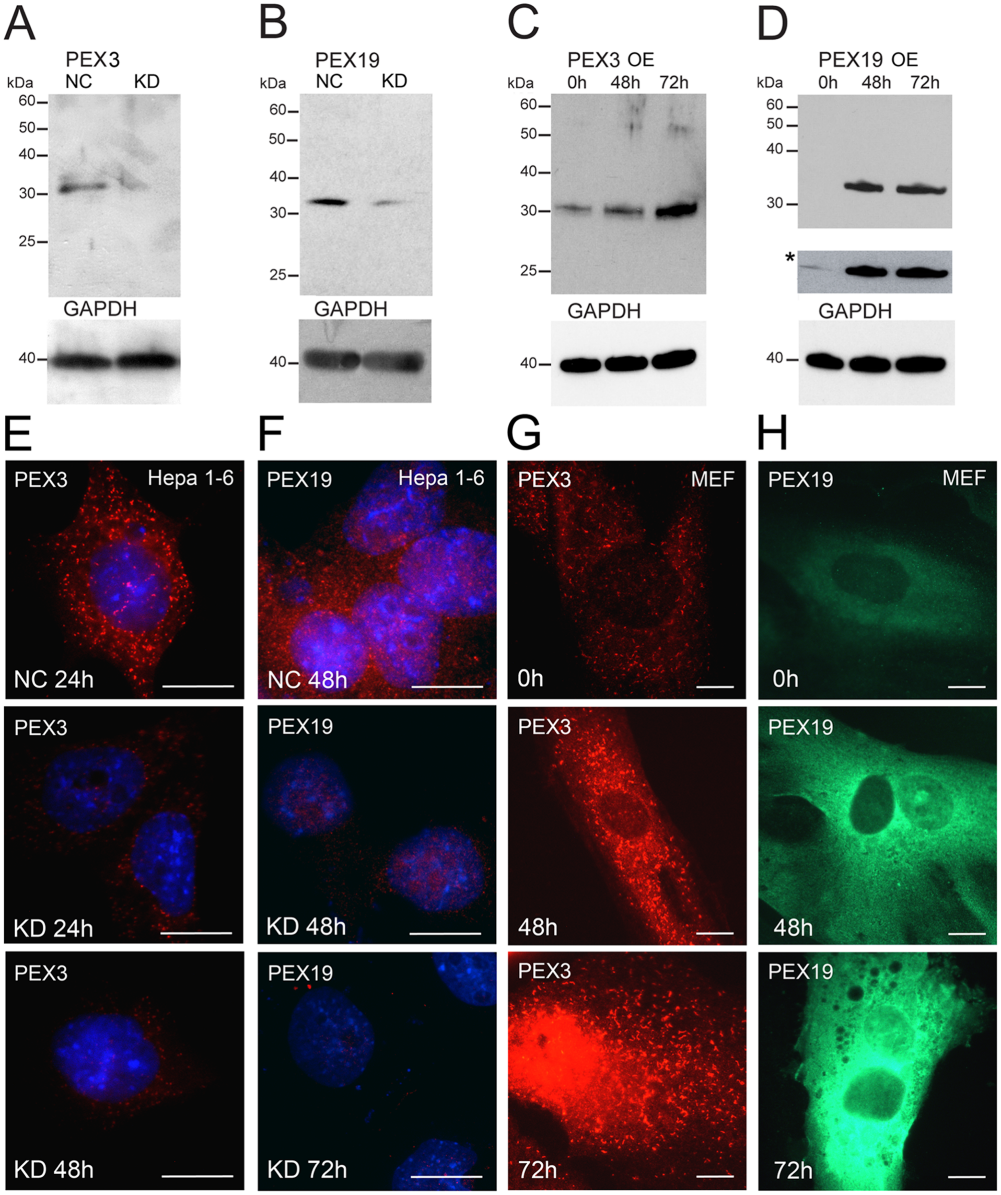
Knockdown and overexpression experiments of *Pex3* and *Pex19* in Hepa 1–6 cells indicate the specificity of our self-generated antibodies. **A and B:** Western blot analysis of Hepa 1–6 cells with and without *Pex3* and *Pex19* gene silencing using the generated antibodies. Hepa 1–6 cells were transfected for 48 h either with 50 nM negative control siRNA (NC) or with 50 nM *Pex3* siRNA (KD) (A) and for 72 h with either 1 μg negative control shRNA (NC) or 1 μg *Pex19* shRNA (KD) (B). **C and D:** Western blot analysis of MEFs overexpressing recombinant PEX3 (C) and PEX19 (D) using the generated antibodies. Lower panel of D indicated with an asterisk (*) represents a longer film exposure. Time point 0 h represents untransfected cells and corresponds to the endogenous level of the analysed peroxins. Protein abundance was analysed after 48 and 72 h after transfection. GAPDH was used as a loading control. **E and F:** Immunofluorescence analysis of Hepa 1–6 cells with *Pex3* (E) and *Pex19* (F) gene silencing using the generated antibodies. “NC” indicates cells transfected with negative control siRNA (E) or negative control shRNA (F), “KD” indicates cells transfected with *Pex3* siRNA (E) or *Pex19* shRNA (F). **G and H:** Immunofluorescence analysis of MEFs overexpressing *Pex3* (G) and *Pex19* (H) using the generated antibodies. **Scale bars** = 7.5 μm.

Before using the self-generated antibodies to investigate the organ distribution of PEX3 and PEX19, we assessed their specificity by Western blot analyses of lysates derived from Hepa 1–6 cells (Fig 2A and 2B) and MEFs (Fig 3C and 3D, time point 0 h). Analyses of the expression of the endogenous PEX3 in Hepa 1–6 cells resulted in the detection of two relatively low abundant bands of about 32 kDa and 53 kDa (PEX3: 372 AA, calculated 40 kDa) (Fig 2A), while in MEFs mainly the band of 32 kDa and a very faint 53 kDa band could be detected (Fig 3C, time point 0 h). Using the newly generated antibody for PEX19, we also obtained two bands of about 35 kDa and 50 kDa (PEX19: 299 AA, calculated 33 kDa) in the Hepa 1–6 cells (Fig 2B), while in MEFs PEX19 was only detectable after longer exposure (Fig 3D, time point 0 h).

Immunofluorescence analysis using the anti-PEX3 antibody in Hepa 1–6 cells and MEFs resulted in a perinuclear-enriched punctuated staining pattern suggesting that the antibody is recognizing the peroxisomal compartment (Fig 2C and 2D). When using the antibody directed against PEX19 in the same cells, we predominantly found a cytosolic staining (Fig 2C and 2D). In Hepa 1–6 cells, the PEX19 antibody also detected some organelle-like punctuated structures, which partially colocalised with the PEX3 staining (Fig 2C). In MEFs, PEX3 and PEX19 did not colocalise, suggesting that in these cells PEX19 is mainly cytosolic (Fig 2D).

We next silenced the gene expression of *Pex3* or *Pex19* in Hepa 1–6 cells (Fig 3A and 3B). MEFs could not be used for these experiments since they did not survive the transfection procedure with either the *Pex3* siRNA or the *Pex19* shRNA. The Western blot results showed a reduction for both PEX3 (65% reduction) and PEX19 (80% reduction) at 48 h and 72 h after transfection, respectively (Fig 3A and 3B).

To complement the results obtained from the Western blotting, we next analysed the knockdown of *Pex3* or *Pex19* in Hepa 1–6 cells by immunofluorescence analysis (Fig 3E and 3F). Following the knockdown of *Pex3*, 34% of counted cells (n = 272) transfected for 24 h displayed drastically reduced PEX3 staining intensity and peroxisome number (Fig 3E). The PEX19 labelling intensity was noticeably weaker in the cytosol and the peroxisomes in 42% of the analysed cells (n = 253) transfected with the *Pex19* shRNA plasmid 48 h after transfection, but without any change in the distribution pattern (Fig 3F).

We next analysed the abundance of the two peroxins in MEFs after transfection with *Pex3* and *Pex19* overexpression plasmids. Protein bands of increasing intensity were detected in the lanes corresponding to the cells overexpressing *Pex3* and *Pex19* at 48 and 72 h post-transfection (Fig 3C and 3D). Compared to the time point 0 h, the transfection produced approximately 3- and 20-fold increases of protein levels for PEX3 and PEX19, respectively. The increase in protein abundance after 48 and 72 h post-transfection was also noticeable in the immunofluorescence stainings (Fig 3G and 3H). Transfection with the *Pex3* overexpression plasmid resulted in an increase of the PEX3 staining intensity on the peroxisomes as well as an increase in the peroxisome number (Fig 3G). Overexpressing *Pex19* in MEFs resulted in an increase in the cytosolic labelling with no association of the staining to peroxisomes (Fig 3H). Taken together these results strongly suggest that the generated antibodies are largely specific for PEX3 and PEX19.

### PEX3 and PEX19 partially colocalise in peroxisomes of Hepa 1–6 cells

**Fig 4:**
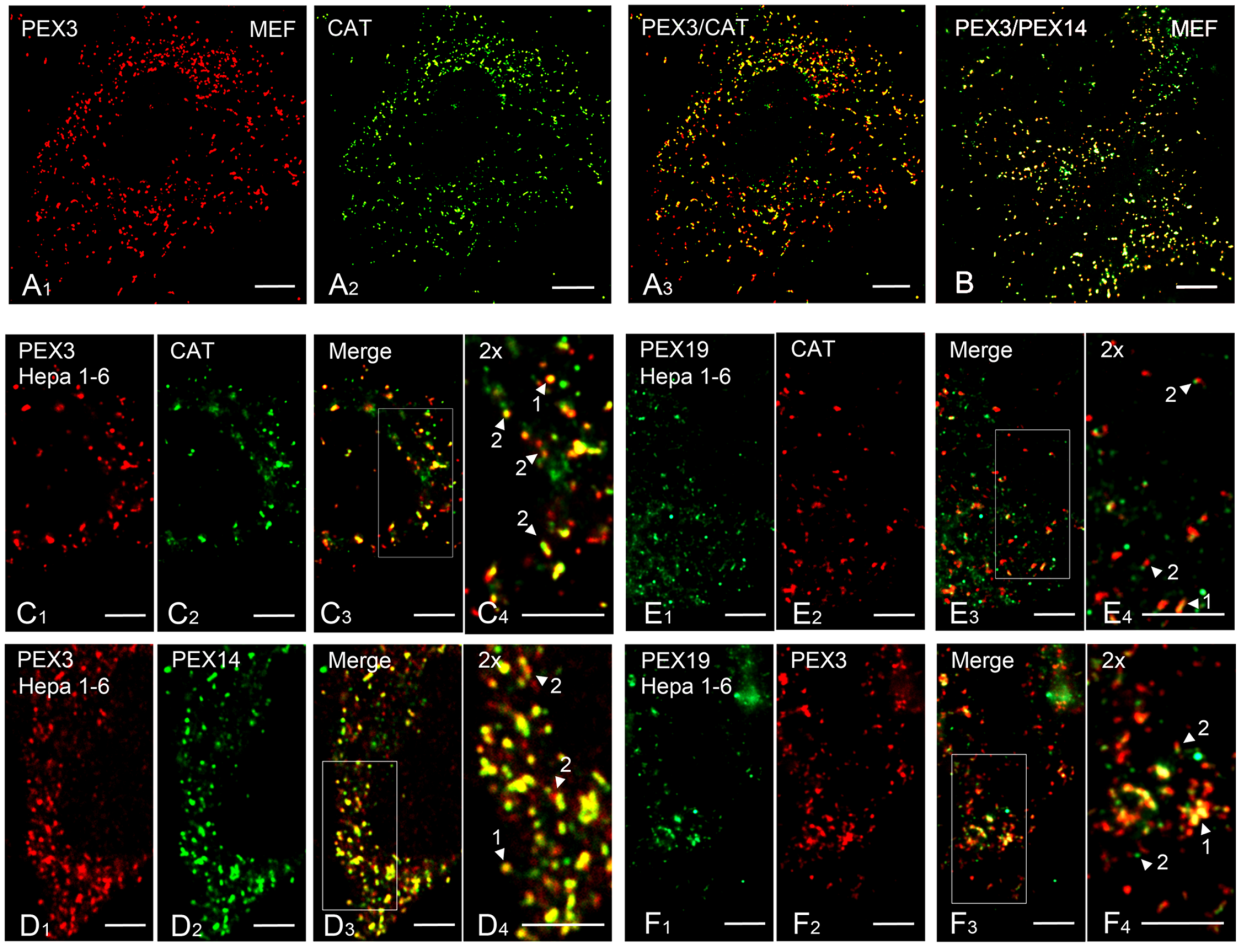
PEX3, PEX19, PEX14 and catalase show heterogeneity in their distribution patterns in the peroxisomal compartment. **A1-3:** Analysis of the colocalisation of PEX3 and CAT in MEFs. **B:** Double staining for PEX3 and PEX14 in MEFs. **C1-4:** Higher magnifications of a double immunofluorescence staining for PEX3 and CAT in Hepa 1–6 cells. C4 represents a 2-fold magnified image of the rectangle marked in C3. **D1-4:** Double immunofluorescence staining for PEX3 and PEX14 in Hepa 1–6 cells. D4 represents a 2-fold magnified image of the rectangle marked in D3. **E1-4:** Higher magnifications of a double immunofluorescence staining for PEX19 and CAT in Hepa 1–6 cells. E4 represents a 2-fold magnified image of the rectangle marked in E3. **F1-4:** Double immunofluorescence staining for PEX19 and PEX3 in Hepa 1–6 cells. F4 represents a 2-fold magnified image of the rectangle marked in F3. **Arrowheads** indicate either complete overlap of the staining (1) or partial overlap (2). **Scale bars** = 7.5 μm.

We were next interested to investigate the exact subcellular localisation of PEX3 and PEX19. For this purpose, we stained Hepa 1–6 cells or MEFs using the antibodies directed against PEX3 and PEX19 in combination with antibodies against the peroxisomal membrane protein PEX14 and the matrix enzyme catalase. While in MEFs, PEX3 colocalised with either catalase (CAT) or PEX14 (Fig 4A and 4B) we noticed a heterogeneous intraperoxisomal distribution in Hepa 1–6 cells.

PEX3 and CAT double labelling produces a complete overlap in 20 ± 5% of the total number of peroxisomes (which here is the sum of all counted peroxisomes labelled with both antibodies as well as with PEX3 and CAT only, n = 2015 peroxisomes) (Fig 4C_1-4_, arrowhead 1). In 11 ± 2% of the total number of peroxisomes PEX3 was located at focal points on top of the more widespread CAT labelling (Fig 4C_1-4_, arrowhead 2). The rest of the labelled peroxisomes either were positive for PEX3 (33 ± 4%) or for catalase (40 ± 3%) only (Fig 4C_1-4_), suggesting variable PEX3 and catalase contents in individual peroxisomes. A complete PEX3/PEX14 overlay was obtained in 22 ± 7% of the total number of peroxisomes (which here is the sum of all counted peroxisomes labelled with both antibodies, as well as with PEX3 and PEX14 only, n = 2265 peroxisomes) (Fig 4D_1-4_, arrowhead 1). In 18 ± 8% of all labelled peroxisomes PEX3 and PEX14 were located at different foci on the same organelle (Fig 4D_1-4_, arrowhead 2). The rest of the labelled peroxisomes either were positive for PEX3 (35 ± 5%) or for PEX14 (38 ± 6%) only (Fig 4D_1-4_).

PEX19 is partially present in the cytosol but is also associated with PEX3 (colocalisation in 36 ± 12% of all counted PEX3-positive peroxisomes, n = 4649 peroxisomes) and CAT (colocalisation in 18 ± 5.8% of all counted catalase-positive peroxisomes, n = 6753 peroxisomes) in Hepa 1–6 cells (Fig 4E_1-4_ and 4F_1-4_ arrowhead 1). These observations suggest a differential subperoxisomal distribution of PEX3, PEX19, PEX14 and CAT.

### Different organs exhibit strong variations in the protein abundance and the molecular weight of PEX3 and PEX19 as well as in the subcellular localisation of PEX19

**Fig 5:**
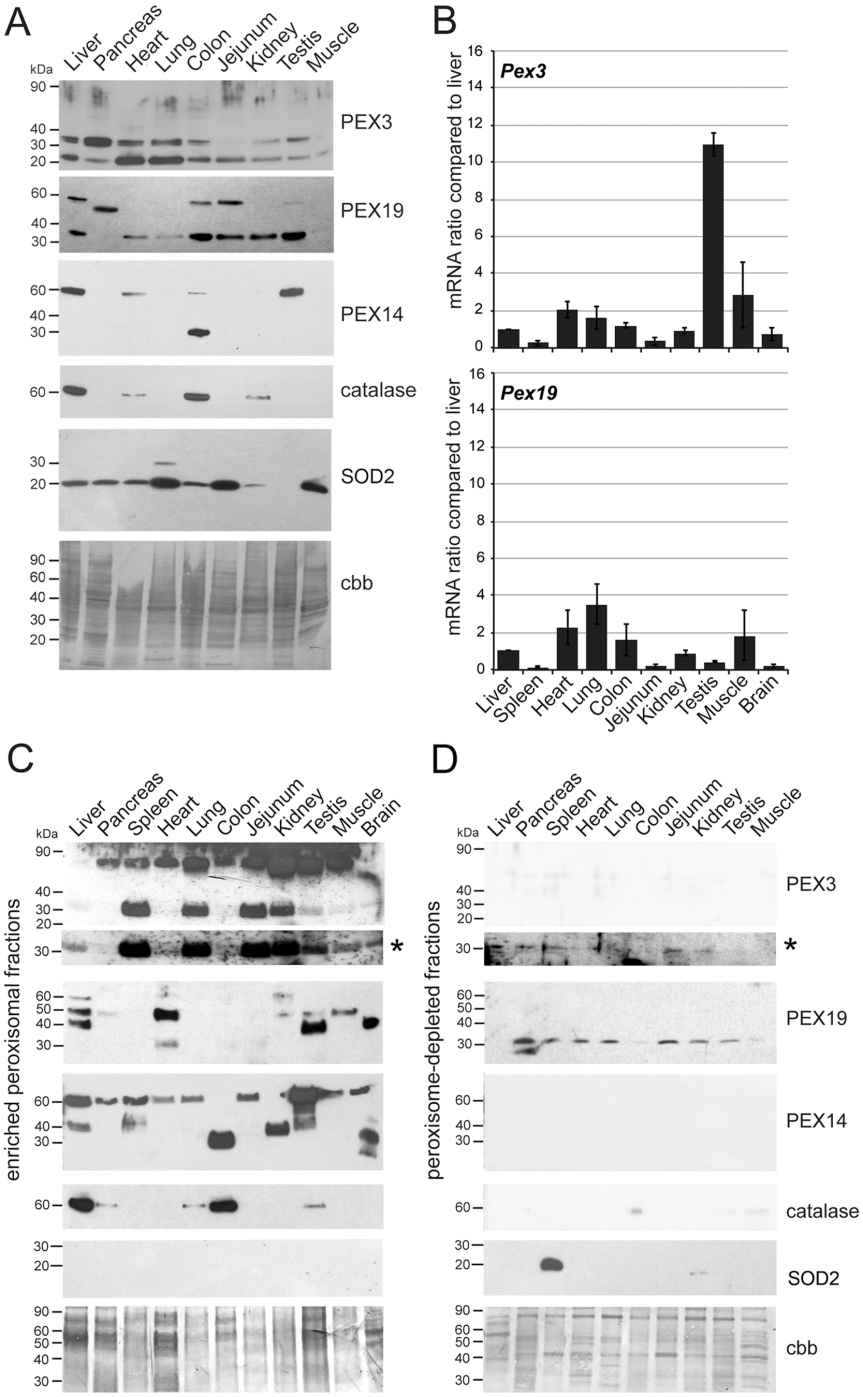
PEX3 and PEX19 proteins and their corresponding mRNAs are not expressed in a concerted manner in different organs. **A:** Western blot analysis of whole tissue lysates detected with the antibodies generated against PEX3 and PEX19 in comparison with the protein abundance of PEX14, CAT and the mitochondrial protein SOD2. Ten μg of protein were loaded per lane. As loading control we have stained the Western blot with CBB. **B:** qPCR analysis of *Pex3* (C) and *Pex19* (D) mRNA levels using the cDNA reversely transcribed from total RNA derived from different mouse organs. Values are expressed as fold-change compared to the expression levels obtained for the liver, which was set to 1. The graphs display calculated mean values (Mean *Pex3*, Mean *Pex19*) from qPCR analyses using three different reference genes (*Ppia*, *Rpl13* and *Tbp*) for normalisation (S1 Fig). The error bars represent the standard deviation of three independent experiments. **C and D:** Western blot analysis of peroxisome-enriched (C) and peroxisome-depleted (D) fractions of different mouse tissues detected with the antibodies generated against PEX3 and PEX19 in comparison with the protein abundance of PEX14, catalase and the mitochondrial protein SOD2. Ten μg of protein were loaded per lane. Panels marked with an asterisk (*) represent longer exposures of the PEX3 Western blots. As loading control we have stained the Western blot with CBB.

We have next estimated the amount of PEX3 and PEX19 in whole lysates derived from different organs. PEX3 was detected in all analysed organs, but was relatively low abundant in the jejunum and skeletal muscle (Fig 5A). All organs also displayed a low molecular weight band of 20 kDa that we cannot allocate (Fig 5A). PEX19 was also detected in all organs, but was extremely low abundant in skeletal muscle (Fig 5A). The molecular weight of the detected bands corresponded to the predicted one of about 33 kDa except for pancreas, which displayed a band of 50 kDa (Fig 5A). Additionally in liver, colon, jejunum and testis a band of 60 kDa was detected (Fig 5A).

To investigate whether the abundance of peroxisomal proteins is regulated concertedly in different organs, we compared the distribution of PEX3 and PEX19 to that of the marker proteins catalase and PEX14. For catalase one single band of 64 kDa was detected in liver, heart, colon and kidney (Fig 5A). By far the highest expression of this enzyme was found in the liver, as expected, and in the colon (Fig 5A). The predicted molecular weight for PEX14 is 34 kDa, however, it previously has been shown that the protein runs at about 60 kDa (57 kDa) in Western blots of liver homogenates [39]. A protein band of approximately 60 kDa can be detected in liver, heart, colon and testis (Fig 5A). In the colon, we additionally detected a smaller band of the predicted molecular weight of approximately 30 kDa. The appearance of two protein bands on Western blot analysis of PEX14 has been already shown in studies using enriched peroxisomal fractions from different areas of the newborn mouse brain [34].

Western blot analysis of the enriched peroxisomal fractions using the anti-PEX3 antibody resulted in the detection of a strong 32 kDa band in spleen, lung, jejunum, kidney and testis (Fig 5C). In liver, skeletal muscle and brain (frontal neocortex) this protein band was less abundant and in pancreas, heart and colon the band was only visible after longer exposure (Fig 5C). In addition to the 32 kDa band, a strong band of 70 kDa could be visualized in most organs except liver and brain (Fig 5C). Opposite to what was previously suggested for purified PEX3 protein, loading the sample without boiling to 95°C in either the presence or the absence of SDS [54,56] did not dissolve the 70 kDa band (data not shown). Also the addition of different concentrations of urea (0.25, 1 and 4 M) did not solubilize the 70 kDa band but instead increased its intensity (data not shown). SOD2 was not detected in any of the organs indicating that the enriched peroxisomal fractions were essentially free from detectable contaminations with mitochondrial matrix proteins.

PEX19 is a soluble protein that is mainly located in the cytosol [57]. To investigate the tissue-specific subcellular localisation of this protein, we therefore used a peroxisome-depleted fraction, containing mainly microsomes and cytosolic proteins, which we obtained during the subcellular fractionation of the whole organ lysates. The Western blot analysis for PEX19 revealed a band of the expected size of approximately 35 kDa in the peroxisome-depleted fraction of all analysed organs except liver (Fig 5D). As expected, using antibodies against PEX3 and PEX14 next to no signal could be detected in the peroxisome-depleted fraction. Labelling of the same fractions using the anti-catalase antibody detected a very faint protein band of the expected molecular weight of approximately 60 kDa in lung, testis and skeletal muscle only after extended exposure of the Western blot membrane (Fig 5D). Also, no SOD2 was detected in the peroxisome-depleted fractions except for the samples derived from spleen, probably a result of mitochondrial lysis during the isolation procedure. This demonstrates that homogenization and differential centrifugation of the organs under our experimental conditions did not result in leakage of the peroxisomes or fragmentation of their membrane.

Although PEX19 is mainly located in the cytosol, a small percentage (5%) was reported to be present in the peroxisomal membrane in rat liver extracts [51]. Western blot analysis using the anti-PEX19 antibody detected several protein bands in the peroxisome-enriched fractions of liver, pancreas, heart, kidney, testis, skeletal muscle and brain (Fig 5C). The predicted size for PEX19 is of approximately 33 kDa, however, like in the whole organ lysate, we detected bands that were larger than expected. In liver, testis and brain a band of approximately 40 kDa was visible (Fig 5C). Additionally, in liver, heart, kidney, testis, and skeletal muscle a band of approximately 50 kDa was detected (Fig 5C). Treatment of the peroxisome-enriched fractions with urea (0.25, 1 and 4 M) prior to loading on the SDS-PAGE did not reduce the number of bands, but instead augmented the intensity of high molecular products suggesting that they are not the result of dimerization of PEX19 (data not shown).

Comparison of the signals obtained for PEX3, PEX19, catalase and PEX14 in the peroxisome-enriched fractions suggests that the amount of these proteins is regulated independently of each other in the different organs (Fig 5A).

### Comparative analysis of the mRNA levels of Pex3 and Pex19 genes in different organs of the adult mice

Because we wanted to compare the *Pex3* and *Pex19* mRNA levels in different organs we used three reference genes for the normalization of the qRT-PCR results. The commonly used reference genes *Gapdh* and *ß*-*actin* (*Actb*) were excluded *a priori* due to the organ-dependent variability of their mRNA level. Among the reference genes, which were shown to be minimally regulated during cell-cycle and nutritional fluctuations and which were proposed for comparison of different organs [58,59], we decided to use *Tbp*, *Rpl13* and *Ppia*. Pancreas was omitted from the quantitative mRNA analyses due to its exceedingly high content of RNases. For the comparison of the expression profiles of *Pex3* and *Pex19* genes in different organs, the ct values were normalized to each of the three reference genes and liver was set to 1 (S1A and S1B Fig; norm *Ppia*, norm *Tbp* and norm *Rpl13*). Next, we calculated the *Pex3* and *Pex19* gene expression as means of the three separately normalized values (Fig 5B). Absolute values for the *Pex3* (S1A Fig) and the *Pex19* (S1B Fig) transcripts normalized with the different reference genes were not equal, but the overall organ distribution was comparable. Independent of the reference gene used for the normalization, by far the highest mRNA level for *Pex3* (Fig 5B) (9-10-fold increase compared to liver) was found in the testis. The mean *Pex3* mRNA level was approximately double in heart, lung and skeletal muscle compared to liver, while the lowest amount was found in spleen and jejunum (Fig 5B). The qRT-PCR analysis further showed that the highest *Pex19* mRNA expression (Fig 5B) was found in the heart, lung, colon and skeletal muscle (2-4-fold increment compared to liver), the lowest in spleen, jejunum, testis and brain. Also for PEX19 the absolute values obtained using *Ppia*, *Tbp* or *Rpl13* for the normalisation varied slightly, but the overall organ distribution was comparable (Fig 5B). Because of the detection of multiple bands on the Western blot analysis we have searched the NCBI database for *Pex19* alternative transcripts. Two isoforms of *Pex19* are annotated in the NCBI database (www.ncbi.nlm.nih.gov/nucleotide). Isoform 1 is a 3131 bp long transcript (the corresponding protein contains 299 aa) in which all predicted exons are expressed. Isoform 2 lacks the first exon and 40 bp of the second exon and is 2855 bp long (the corresponding protein contains 207 aa). The 3´ and 5´untranslated regions are identical. We designed *Pex19* isoform 1 specific qPCR primers to investigate the percentage of *Pex19* isoform 1 to the total *Pex19* mRNA expression. The results show that in Hepa 1–6 cells and MEFs both isoforms are equally expressed. In all analysed organs except liver the expression of isoform 1 predominated (>65%). In spleen, lung, testis and skeletal muscle only isoform 1 was expressed (S6 Table). Thus, the expression of two PEX19 protein isoforms or differences in their ratio is probably not the cause for the differently sized bands detected by Western blot analysis.

### The abundance of PEX3 and PEX19 proteins varies between the different organs

From older studies it is well known that peroxisomes are very numerous and easily detectable in liver and kidney using antibodies against catalase, ABCD3 and PEX14 (for an overview see [30,39]). To investigate the organ- and cell type-specific distribution of the peroxins PEX3 and PEX19 we used immunofluorescence stainings on formalin-fixed paraffin-embedded organ sections. For each organ, consecutive sections and comparable areas were photographed and colocalisation studies were performed by using the peroxisomal marker proteins PEX14, together with PEX3, and ABCD3 together with PEX19. During these experiments we noticed that i) labelling of PEX3 in paraffin sections was difficult in comparison to the one of other peroxisomal biogenesis proteins such as PEX14 due to its low abundance and ii) it was difficult to visualize the peroxisome-bound form of PEX19 by fluorescence microscopy in tissues in which the cytosolic-associated form was strongly labelled. To overcome these two problems we have used very thin tissue sections (approximately 2 **μ**m), adjusted the tissue permeabilization protocol for each organ determined the best dilution for both anti-PEX3 and anti-PEX19 antibodies in serial dilutions and optimized the microscope settings for the areas of interest (S5 Table). The antibody concentration-range was chosen according to pre-existing information derived from our Western blot analysis on peroxisome-enriched fractions (Fig 5C) and immunofluorescence stainings of MEFs and Hepa 1–6 cells (Fig 2C and 2D) as well as from known concentration ranges used for immunofluorescence experiments of the antibodies against PEX14, catalase and ABCD3. After careful evaluation of all specimens, an average concentration of the antibodies with which all the organs could be fairly well stained was used to compare the relative abundance of PEX3 (1:500) and PEX19 (1:10,000) in distinct organs and the images were taken with a Leica DM RD fluorescence microscope using the same camera settings. The highest staining intensity for PEX3 was found in kidney and testis followed by skeletal muscle, heart, jejunum and colon > pancreas > liver > lung. In contrast, cytoplasmic PEX19 was most abundant in kidney and pancreas followed by skeletal muscle, heart > testis > liver > colon and jejunum > lung (S2 Fig). Even in the tissue specimens with highest stainings intensities for PEX3 and PEX19 (e.g. kidney or testis) appropriate negative controls were almost devoid of background staining (S2 Fig).

In the following, we will describe the cell type-specific distribution and subcellular localisation of PEX3 and PEX19 analysed by immunofluorescence staining using antibody dilutions and microscope settings individually optimized for each organ.

#### 1. Kidney

**Fig 6:**
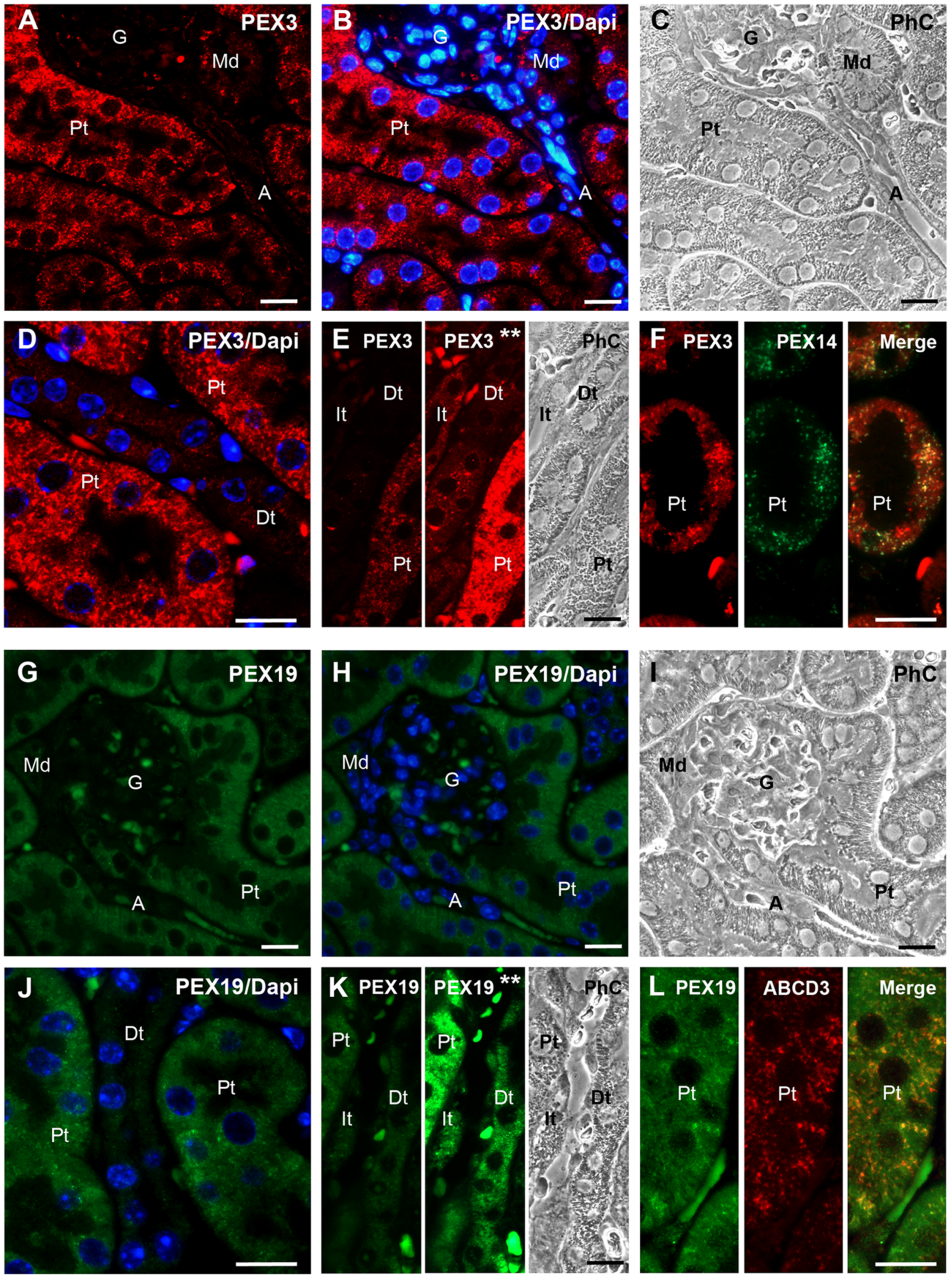
PEX3 and PEX19 are highly abundant in the proximal tubules of the kidneys and display a peroxisomal (PEX3) and dual peroxisomal/cytosolic (PEX19) localisation. **A and B:** PEX3 staining of proximal tubules and of the glomerulum with its juxtaglomerular apparatus (e.g. macula densa) in the renal cortex. **C:** Phase-contrast image of the same area shown in Figs A and B. **D:** Comparison of PEX3 abundance in proximal and distal tubules at higher magnification. **E:** Comparison of PEX3 abundance in proximal, distal and intermediate tubules. The three stripes represent: left—normal exposure time; middle– 2-fold augmented brightness; right—phase-contrast image. **F:** Colocalisation of PEX3 and PEX14 in a proximal tubule. **G and H:** PEX19 staining in proximal tubules and glomerulum with the associated macula densa of the distal tubule. **I:** Phase-contrast image of the same area shown in Figs G and H. **J:** Comparison of PEX19 abundance in proximal and distal tubules at higher magnification. **K:** Comparison of PEX19 abundance in proximal, distal and intermediate tubules. The three stripes represent: left—normal exposure time; middle—2-fold digitally augmented brightness; right—phase-contrast image. **L:** Colocalisation of PEX19 and ABCD3 in a proximal tubule. **Nuclear staining:** in Figs B, D, H and J with Hoechst 33342. **Abbreviations:** G, glomerulum; Md, macula densa (part of the distal tubule); Pt, proximal tubule; Dt, distal tubule; A, artery; It, intermediate tubule; PhC, phase-contrast; Asterisks (**), 2-fold digitally augmented brightness. **Scale bars** = 15 μm.

In the kidney, we analysed the distal (Dt), proximal (Pt) and intermediate (It) tubules as well as the glomeruli (G) and the macula densa (Md). For both, PEX3 and PEX19, the highest fluorescent signal was found in the proximal tubules (Fig 6A, 6D, 6G and 6J), while only a very weak fluorescent signal could be visualized in all other parts of the nephron (Fig 6A, 6D, 6E, 6G, 6J and 6K). This corresponds well with previous reports in which the strongest staining for catalase and PEX14 was observed in the proximal tubules [30,39]. Inside the epithelial cells of the distal and proximal tubules, PEX3 was found in large round or tubular structures. Double staining with antibodies against PEX14 confirmed that the antibody for PEX3 stains peroxisomes, however, individual organelles were labelled with different intensity in comparison to PEX14, suggesting different subpopulations of peroxisomes (Fig 6F) as noticed previously in Hepa 1–6 cells (Fig 4).

Albeit the distribution of PEX19 in different tubules of the nephron was similar to that observed for PEX3 (Fig 6G–6L), PEX3 was located exclusively in the peroxisomes of the epithelial cells of the proximal tubule, while the staining for PEX19 was associated with the cytoplasm as well as with peroxisomes, which were most prominently labelled in the proximal tubules (Fig 6J). The overlay with the staining of ABCD3 confirmed that PEX19 is indeed bound to peroxisomes in this cell type (Fig 6L). Cytosolic- and peroxisome-bound PEX19 could also be identified in the distal and the intermediate tubules using longer exposure times (Fig 6K).

#### 2. Testis

**Fig 7:**
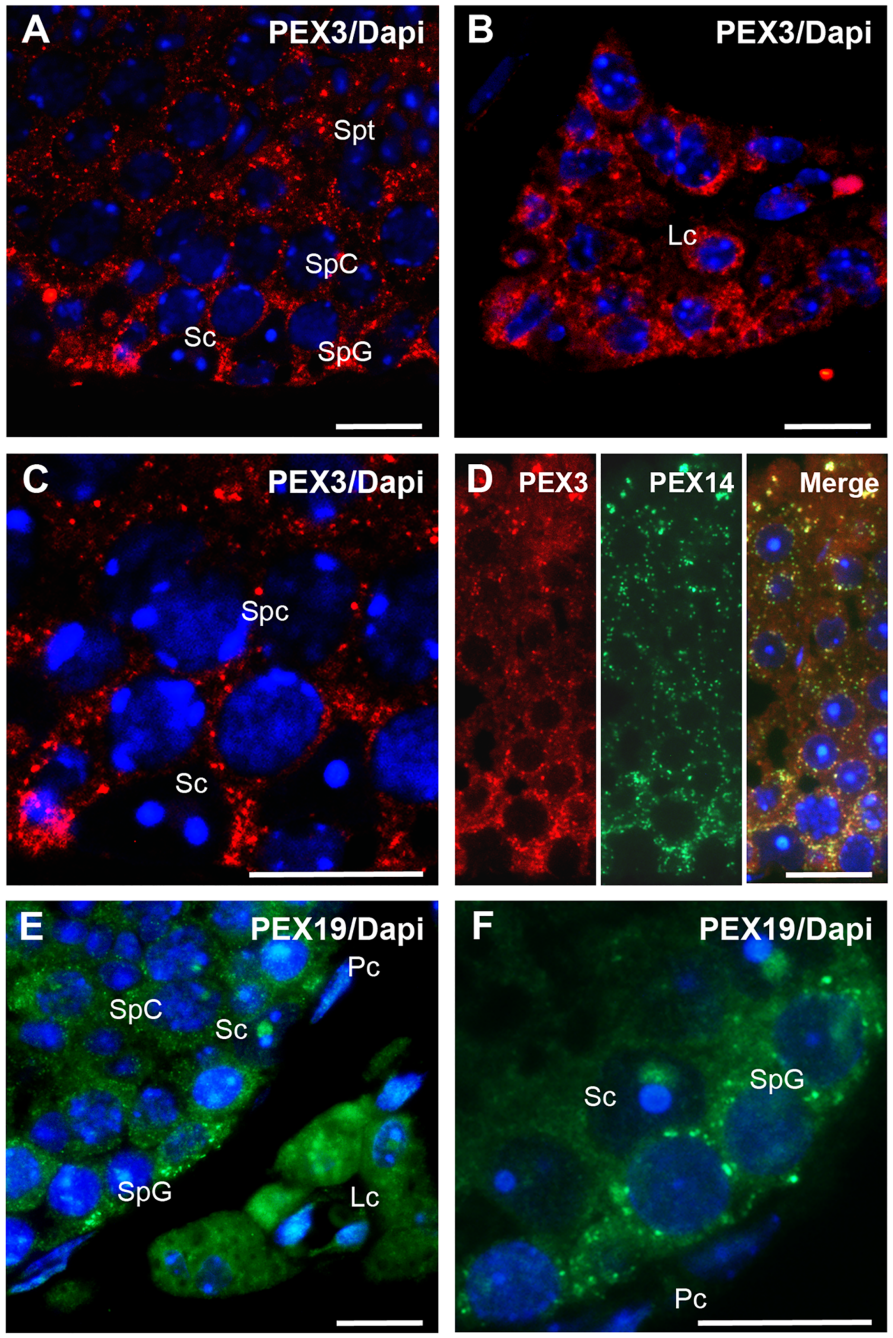
PEX3 and PEX19 are highly abundant in the testis and are present in germ and somatic cells. **A:** Overview of the PEX3 staining in germ and Sertoli cells of a seminiferous tubule. **B:** Distribution of PEX3 in Leydig cells. **C:** Higher magnification of PEX3-labelled peroxisomes in the basal part of a seminiferous tubule. **D:** Colocalisation of PEX3 and PEX14 in the germinal epithelium. **E:** Overview of the PEX19 staining in different cell types of a seminiferous tubule and interstitial Leydig cells. **F:** Higher magnification of PEX19 staining of the basal layer of the germinal epithelium. **Nuclear staining:** in Figs A-F with Hoechst 33342. **Abbreviations:** Sc, Sertoli cells; SpG, spermatogonia; SpC, spermatocytes; Spt, spermatids; Lc, Leydig cells; Pc, peritubular cells. **Scale bars** = 15 μm.

In comparison to kidney, PEX3 was expressed at similarly high levels in almost all testis-specific cell types. As shown in Fig 7A, strong labelling was observed in all germ cells such as spermatogonia (SpG), spermatocytes (SpC) and spermatids (Spt) as well as in the somatic cell types (Sertoli cells, Sc, and Leydig cells, Lc) (Fig 7A and 7C; Sc and Fig 7B; Lc) except in the peritubular cells (Pc). As shown by the double staining, the distribution of PEX3 in the germinal epithelium was identical to the one of PEX14 (Fig 7D).

Staining of the germinal epithelium with the antibody for PEX19 resulted in a similar distribution pattern than that observed for PEX3. Cytosolic PEX19 is highly abundant in all germ cells of the seminiferous tubules and peroxisome-bound PEX19 is particularly abundant in spermatogonia (Fig 7E; SpG). In the somatic cells, the staining for PEX19 is very strong in Leydig cells followed by Sertoli cells (Fig 7E and 7F; Lc and Sc), while it is not detectable in peritubular cells (Fig 7E and 7F; Pc).

#### 3. Liver

**Fig 8:**
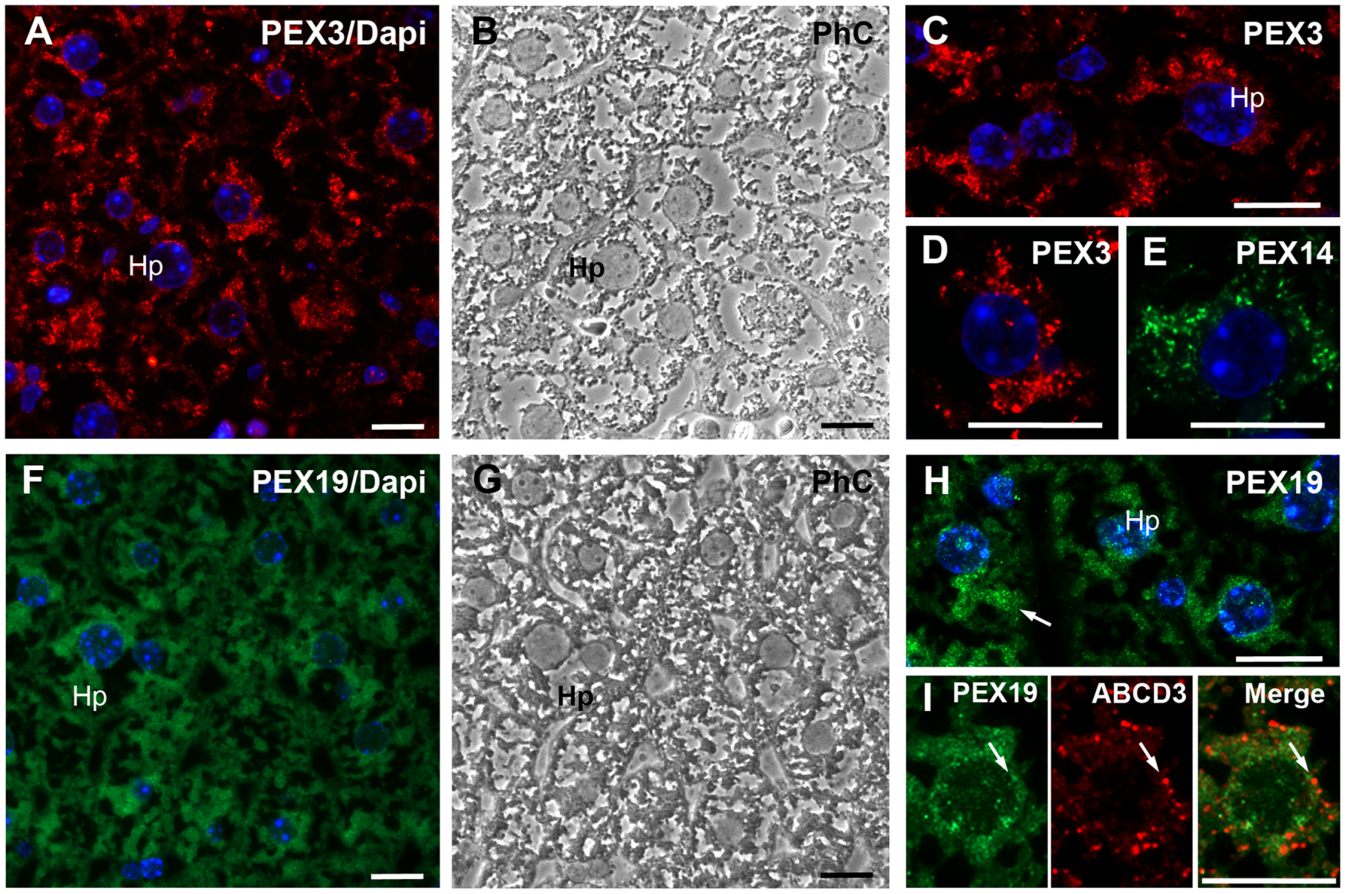
Hepatocytes in the liver display a relatively low abundance of PEX3 and PEX19 compared to the one of PEX14 and ABCD3. **A:** Overview of the PEX3 staining in peroxisomes in hepatocytes of the periportal region in the liver. **B:** Phase-contrast of the image shown in A. **C:** Higher magnification of PEX3-labelled peroxisomes in periportal hepatocytes. **D and E:** Comparison of the peroxisomal pattern in hepatocytes in stainings with the anti-PEX3 (D) or anti-PEX14 (E) antibodies. **F:** Overview depicting the PEX19 distribution in a liver region comparable to A. **G:** Phase-contrast of the image shown in F. **H:** Higher magnification image of PEX19-labelled peroxisomes in hepatocytes (marked with arrow). **I:** Comparison of the PEX19 and ABCD3 subcellular localisation in hepatocytes. Arrows indicate partial overlap of PEX19 and ABCD3. **Nuclear staining:** in Figs A, C, D, E, F, H, I with Hoechst 33342. **Abbreviations:** Hp, hepatocytes, PhC, phase-contrast. **Scale bars** = 15 μm.

Since liver contains a large number of peroxisomes, we also expected high PEX3 content. However, as already predicted from the Western blot results (Fig 5A and 5C), the PEX3 antibody only weakly stained round and tubular peroxisomes as well as large granular/patchy clusters of peroxisomes in the hepatocyte´s cytosol that are typical for these cells (Fig 8A–8C; Hp). Comparison of the individual stainings of PEX3 and PEX14 revealed a similar subcellular distribution pattern (Fig 8D and 8E).

In liver, the anti-PEX19 antibody generated a cytosolic staining (Fig 8F–8H) that did not colocalise with the staining of ABCD3 (Fig 8I) and differed from the one observed for PEX3 or PEX14 (Fig 8D and 8E). In addition to the cytosol the antibody directed against PEX19 also stained the hepatocyte´s nucleus with a dotted pattern the nature of which is not clear yet.

#### 4. Pancreas

**Fig 9:**
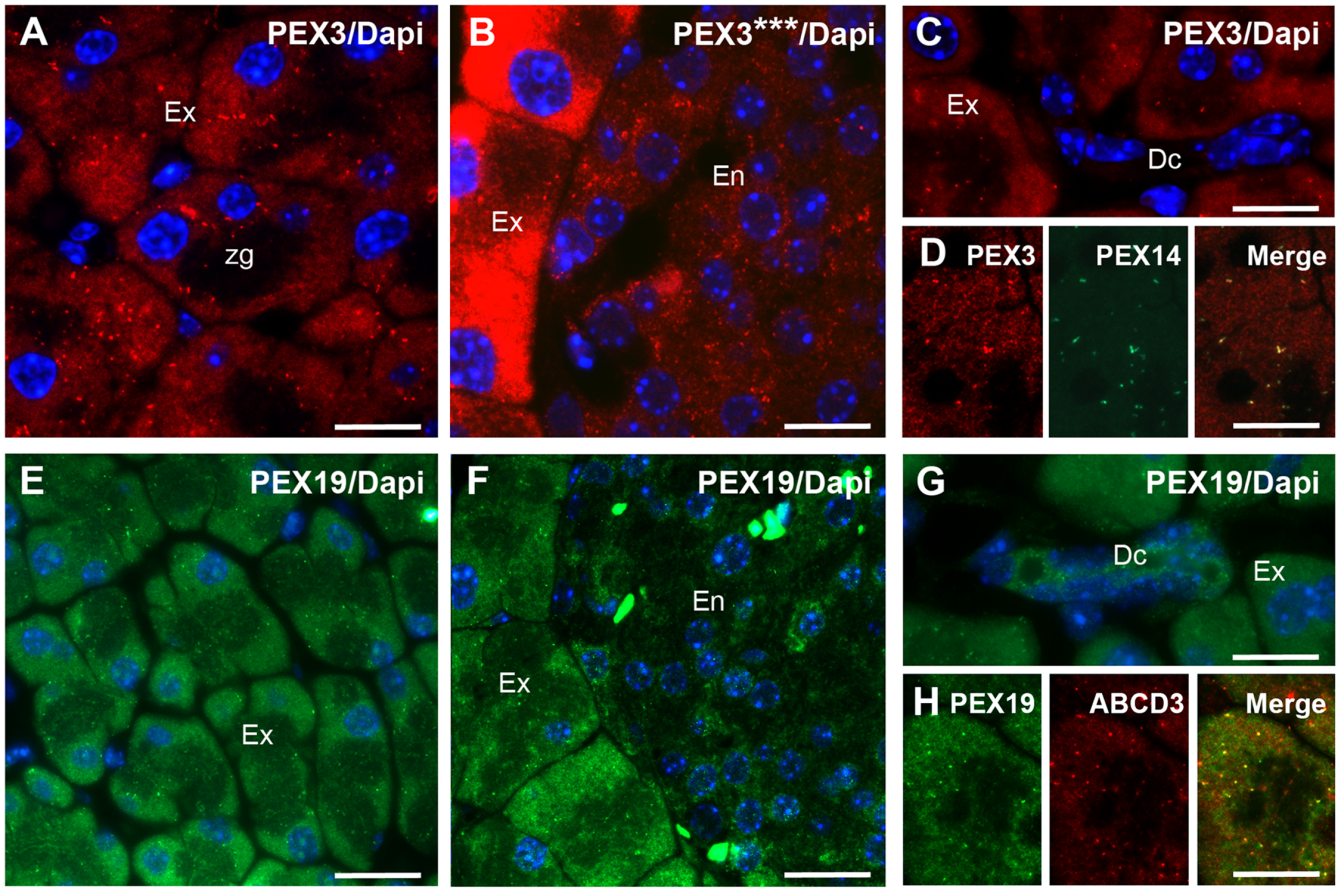
In the exocrine pancreas PEX3 and PEX19 are associated with peroxisomes. **A:** Subcellular localisation of PEX3 in acinar cells of the exocrine pancreas. **B:** Comparison of the abundance of PEX3 in exocrine and endocrine pancreas after 3-fold augmentation of the brightness (***). **C:** PEX3 in an exocrine intercalated duct. **D:** Colocalisation of PEX3 and PEX14 in acinar cells of the exocrine pancreas. **E:** Subcellular localisation of PEX19 in acinar cells of the exocrine pancreas. **F:** Comparison of the abundance of PEX19 in exocrine and endocrine pancreas. **G:** PEX19 in an exocrine intercalated duct. **H:** Colocalisation of PEX19 and ABCD3 in an acinar cell of the exocrine pancreas. **Nuclear staining:** in Figs A-C and E-G with Hoechst 33342. **Abbreviations:** Ex, exocrine pancreas; En, endocrine pancreas; zg, zymogen granules; Dc, exocrine intercalated duct; PhC, phase-contrast; Asterisks (***), 3-fold digitally augmented brightness. **Scale bars** = 15 μm.

We next analysed the distribution of PEX3 in the endocrine (En) (islets of Langerhans) and the exocrine (Ex) part of the pancreas. In exocrine cells, PEX3-positive peroxisomes appeared as heterogeneously distributed, dispersed single spots and tubules (Fig 9A) that colocalised with PEX14 (Fig 9D). The apically located zymogen granules, appeared dark in the immunofluorescence pictures and were not stained for PEX3 (Fig 9A). Similarly to what was previously described for catalase [39], the PEX3 staining was more intense in the exocrine than in the endocrine part (Fig 9B). Indeed, the PEX3 staining of the endocrine pancreas only became visible when the images were overexposed (Fig 9B). This pattern is opposite to the staining pattern that was observed by Grant *et al*. for PEX14 [39], which was more intense in the endocrine than in the exocrine part of the pancreas. In the mentioned study, catalase was, however, only sporadically detected in the islets of Langerhans in what appeared to be endothelial cells. Although the staining for PEX3 in the endocrine part is less intense, it can be detected throughout the majority of cells of the islet of Langerhans. In the epithelium of the excretory intercalated duct, PEX3 was almost not detectable (Fig 9C), similarly to what was previously described for PEX14 [39].

The distribution of PEX19 within the pancreas was comparable to the one of PEX3: a sporadic distribution of PEX19-labelled peroxisomes that colocalised with the ABCD3 staining could be seen in the exocrine part (Fig 9E and 9H), while the staining for the endocrine part was noticeably weaker (Fig 9F). Interestingly, different to the results obtained for PEX3 and PEX14, we found an elevated level of PEX19 associated with the excretory intercalated duct similarly to what was previously described for the catalase staining [39] (Fig 9G).

#### 5. Jejunum and colon

**Fig 10:**
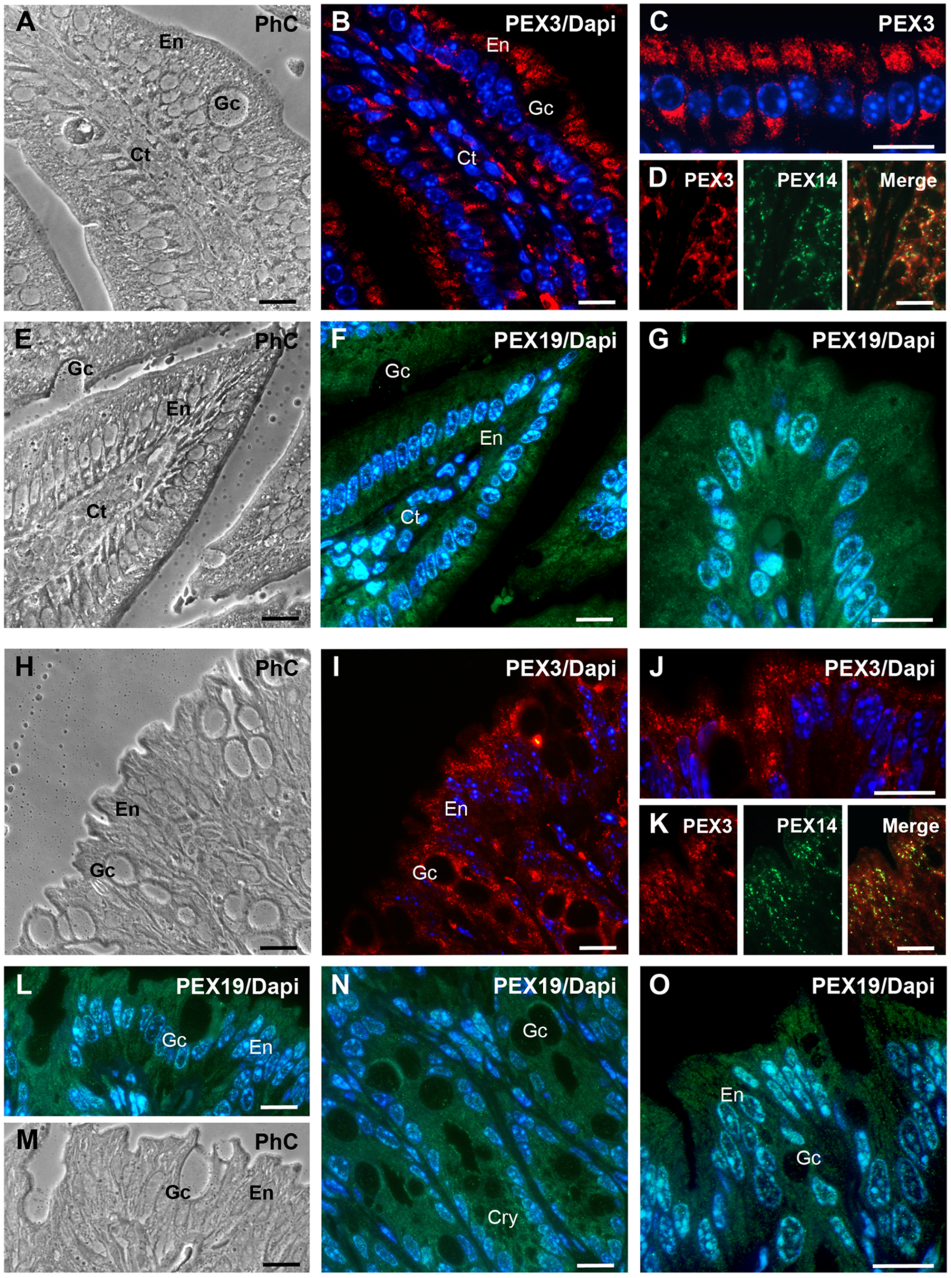
PEX3 is peroxisomal and PEX19 is mainly cytosolic in enterocytes of the jejunum and the colon. **A-O:** Subcellular distribution of PEX3 and PEX19 in the jejunum (A-G) and the colon (H-O). **A:** Phase-contrast image of the villus region shown in B for the immunofluorescence staining. **B:** PEX3 subcellular localisation in a villus of the jejunum. **C:** Higher magnification of PEX3-labelled peroxisomes in enterocytes. **D:** Colocalisation of PEX3 and PEX14 in peroxisomes of enterocytes. **E:** Phase-contrast image of the villus region shown in F for the immunofluorescence staining. **F:** Subcellular localisation of PEX19 in a villus of the jejunum. **G:** Higher magnification of PEX19-positive enterocytes on the tip region of an intestinal villus. **H:** Phase-contrast image of the colon region used for the immunofluorescence staining in I. **I:** Distribution of PEX3 in the epithelium of the colon. **J:** Higher magnification of PEX3-labelled peroxisomes in colonic enterocytes and goblet cells. **K:** Colocalisation of the PEX3 and PEX14 in colonic enterocytes. **L:** Subcellular localisation of PEX19 in the epithelium of the colon. **M:** Phase-contrast image of the region shown in L. **N:** Higher magnification of the PEX19 staining in the epithelium of the colonic crypts. **O:** Higher magnification of PEX19-stained enterocytes of the colon. **Nuclear staining:** in Figs B, C, F, G, I, J, L, N, O with Hoechst 33342. **Abbreviations:** En, enterocyte; Gc, goblet cell; Ct, connective tissue; Cry, crypts; PhC, phase-contrast. **Scale bars** = 15 μm.

In both jejunum (Fig 10A–10D) and colon (Fig 10H–10K), PEX3 was clearly detectable in enterocytes (En), goblet cells (Gc) and cells of the loose connective tissue of the lamina propria (Ct). The staining for PEX3 within the enterocytes was, analogously to the one of PEX14, mainly traced to peroxisomes located apically and basally close to the nucleus (Fig 10C, 10D, 10J and 10K). Generally, the PEX3 staining appeared to be slightly less strong in the colon in comparison with the jejunum. This result is reflected by the Western blot analysis shown in Fig 5C.

The PEX19 staining, either in the jejunum (Fig 10E–10G) or the colon (Fig 10L–10O), was clearly found in the cytosol and the nucleus of enterocytes, goblet cells and cells of the connective tissue of the lamina propria (Fig 10F, 10G, 10N and 10O). The cell type-specific distribution was similar to the one observed for PEX3.

#### 6. Heart and skeletal muscle

**Fig 11:**
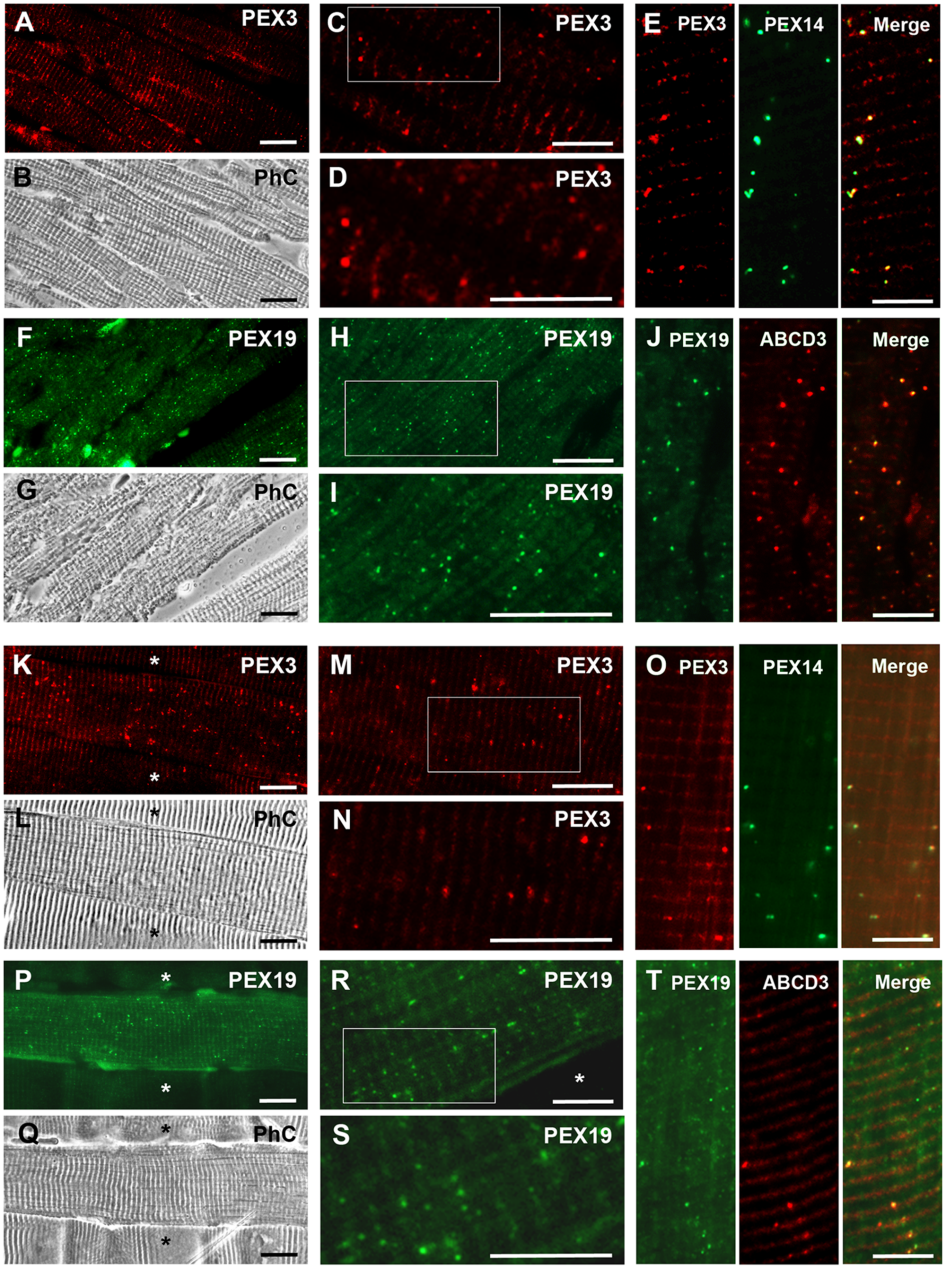
PEX3 and PEX19 are associated with peroxisomes in cardiac and skeletal muscle. **A-T:** Subcellular distribution of PEX3 and PEX19 in cardiac muscle (A-J) and in skeletal muscle (K-T). **A:** Subcellular localisation of PEX3 in cardiomyocytes. **B:** Phase-contrast of the image shown in A. **C:** Higher magnification of a cardiomyocyte showing PEX3-labelled peroxisomes. **D:** 2-fold magnification of the marked area of image C. **E:** Colocalisation of PEX3 and PEX14 in heart muscle. **F:** Cardiomyocytes stained with the PEX19 antibody. **G:** Phase-contrast of the image shown in F. **H:** Higher magnification of cardiomyocytes stained with the PEX19 antibody. **I:** 2-fold magnification of the marked area of image H. **J:** Double-staining of PEX19 and ABCD3. **K:** Distribution of PEX3 in skeletal muscle. Note the difference in staining intensities between individual muscle fibers (*) **L:** Phase-contrast of the image shown in K. **M:** Higher magnification of a skeletal muscle fiber stained for PEX3. **N:** 2-fold magnification of the area marked in Fig M. **O:** Double-staining of PEX3 and PEX14 in a skeletal muscle fiber. **P:** Subcellular localisation of PEX19 in muscle fibers. The difference in labelling intensities between individual muscle fibers is also present and very obvious with regards to the PEX19 staining (*). **Q:** Phase-contrast of the image shown in P. **R:** Higher magnification of a muscle fiber stained for PEX19. **S:** 2-fold magnification of the marked area depicted in R. **T:** Colocalisation of PEX19 and ABCD3 in skeletal muscle. **Abbreviations:** PhC, phase-contrast; Asterisk (*) muscle fibers with very low PEX3 or PEX19 labelling. **Scale bars** = 15 μm.

Results from our laboratory demonstrated larger protein amounts of PEX14, catalase and ABCD3 in the cardiac muscle of the left ventricle than in other parts of mouse hearts suggesting more peroxisomes in this area [60]. We therefore analysed the distribution of PEX3 (Fig 11A–11E) and PEX19 (Fig 11F–11J) in paraffin-embedded sections in the cardiac muscle of the left ventricle. For comparison, we investigated the distribution of both proteins also in skeletal muscle fibers (Fig 11K–11O and 11P–11T). The anti-PEX3 antibody produced a sporadic dotted staining pattern that was mainly located between the myofibrils (Fig 11A and 11K) and colocalised well with the one of PEX14 (Fig 11E and 11O). The anti-PEX19 antibody strongly stained the peroxisomal compartment in cardiomyocytes (Fig 11F–11I) and skeletal muscle fibers (Fig 11P–11S) as confirmed by the overlay with ABCD3 (Fig 11J and 11T). Comparison of the PEX19 staining in cardiac and skeletal muscle revealed that the identification of individual peroxisomes positive for PEX19 was much easier in cardiomyocytes than in skeletal muscle fibers. In the skeletal muscle the cytosolic staining for PEX19 was more prominent and partially masked the peroxisomal one.

Noticeable, we found that the individual skeletal muscle fibers displayed differences in the staining intensities for PEX3 and PEX19 (Fig 11K and 11P). This was particularly evident in the PEX19 stainings (Fig 11P). Using the PEX14 antibody, we confirmed that in the less strongly labelled skeletal muscle fibers the number of peroxisomes was indeed lower. Our data suggest that the two distinct fiber types of the skeletal muscle exhibit a different peroxisomal content.

#### 7. Lung

We previously showed that lung alveolar type II cells and club cells contain the highest number of peroxisomes, while they are markedly lower abundant in alveolar type I cells [36,37,61]. Here, we have investigated the distribution of PEX3 and PEX19 in the lung and found that both proteins were detectable in the epithelial cells of the respiratory bronchioles as well as in alveolar type II cells (S3B, S3C, S3F and S3G Fig). Peroxisomes were clearly visualized in alveolar type II cells with the anti-PEX3 antibody (S3D Fig, arrow), whereas the anti-PEX19 antibody mainly stained the cytosol and peroxisomes were hardly visible (S3H and S3I Fig, arrows). In comparison to alveolar type II cells, we found that PEX19 was more abundant in club cells (notice that the picture shown in S3G Fig is 3 times less exposed compared to the one in S3F Fig). Alveolar type I and endothelial cells were only weakly stained using either of the two antibodies (S3B and S3F Fig).

#### 8. Brain

Peroxisomes are particularly highly abundant in some areas (hippocampus, cerebellum, neocortex) and cell types of the brain (e.g. pyramidal neurons of the motor cortex [34]). Therefore, we mainly focussed our analysis for detecting PEX3 and PEX19 in the brain to this region and have planned the further characterisation of the PEX3 and PEX19 distribution in other brain areas in the near future. Our Western blot analysis of the frontal neocortex (Fig 5C) already revealed the high abundance of PEX14 and PEX19, whereas the one of PEX3 was only weak (Fig 5C). Our immunofluorescence analysis confirmed these findings (S4 Fig). The localisation of PEX3 in the brain was rather difficult. Even in the pyramidal neurons of the primary motor cortex, peroxisomes were only weakly labelled for PEX3 and very long exposure times were necessary to visualize these organelles (S4A and S4B Fig). The clear PEX14 staining of the peroxisomes of the neuropil (S4C Fig) could not be achieved using the anti-PEX3 antibody (S4B Fig) due to the low abundance of this protein in the neuronal processes.

The immunofluorescence staining for PEX19 appeared to be much stronger than the one observed for PEX3. PEX19-positive pyramidal neurons could be visualised in the fluorescence microscope already at lower magnification (S4D Fig). Inside the perikaryon, both cytosolic and peroxisomal staining could be observed, hinting to a double localisation of PEX19 (S4E and S4F Fig). Interestingly, mainly the cytosol around the nucleus of the perikaryon was stained, where also most of the peroxisomes are localised.

## Discussion

PEX3 and PEX19 are indispensable peroxins, which coordinate the early steps of peroxisomal biogenesis. Depletion of these peroxins, independently of each other, causes complete loss of peroxisomal function [6,11,47,48]. The purpose of this study was to provide a comprehensive overview on the organ- and cell type-specific distribution and subcellular localisation of the peroxins PEX3 and PEX19 in mice. We first generated antibodies against both proteins and proved their specificity using knock-down and overexpression experiments. Based on the stainings with these antibodies, our results sustain the hypothesis that the abundance of PEX3 and PEX19 might influence organ-specific characteristics of peroxisomes (number, size and proliferation rate). For PEX19, the regulation of its subcellular localisation could be an additional factor that affects peroxisome biogenesis, maybe by controlling the shuttling frequency of PMPs to the peroxisomal membrane. We further showed that the abundance of the peroxins PEX3, PEX19 and PEX14, of the membrane transporter ABCD3 and of the matrix enzyme catalase is not necessarily regulated in a concerted manner, highlighting that peroxisomal biogenesis, number, enzymatic content and metabolite transporter composition are regulated independently of each other.

### PEX3 and PEX19 are differentially expressed in mouse tissues

Peroxisomes are ubiquitous organelles but are particularly abundant in hepatocytes or the proximal tubules of the kidneys. Because of the close interaction between PEX3 and PEX19, which is necessary for the development of functional mature peroxisomes, at the beginning of this study we speculated i) that organs containing high number of peroxisomes or large quantities of catalase (e.g. liver and kidney) would also contain the largest amounts of PEX3 and PEX19 and ii) that the expression of these closely interacting peroxins would be concertedly regulated. We have therefore compared the distribution of PEX3 and PEX19 to each other and to PEX14, ABCD3 and catalase, which all are well established and frequently used peroxisomal markers [30,39]. PEX14 is part of the peroxisomal membrane-docking complex that is required for the translocation of matrix proteins [1] and is inserted by PEX3 and PEX19 into the peroxisomal membrane [10,62,63]. PEX14 has been shown to be an optimal marker for the visualisation of the number of peroxisomes within individual cells and for comparison of the peroxisomal compartment of different cell types due to its high and constant abundance within the peroxisomal membrane [39]. In Table 1 we have summarised our findings for PEX3 and PEX19 with respect to the staining intensities and subcellular localisation in organ specific cell types (for a comparison to PEX14 see S7 Table). Table 1 exemplifies the highly divergent distribution of these peroxins in mouse organs. The intracellular levels of PEX3 and PEX19 are neither always linked to the number of peroxisomes inside a particular cell type nor is their amount always regulated synchronically. For example, in hepatocytes that notoriously contain large amounts of peroxisomes (stained strongly for catalase, ABCD3 and PEX14) neither PEX3 nor PEX19 where highly expressed as shown by the Western blotting, qRT-PCR and immunofluorescence analysis. The opposite was true for heart and skeletal muscle. In comparison to hepatocytes, both cardiac and skeletal myocytes do neither possess such a large number of peroxisomes [60,64–66] nor contain high levels of the marker enzyme catalase [67]. Nevertheless, in myocytes we found high levels of the PEX3 and PEX19 coding mRNAs and protein. In other cell types containing a large number of peroxisomes, such as Leydig cells in the testis or epithelial cells of proximal tubules in the kidney, also the abundance of PEX3 and PEX19 was high. Interestingly, the abundance of PEX3 and PEX19 does not only vary in relation to catalase or peroxisomal abundance, but also in relation to each other. In hepatocytes and epithelial cells of the excretory duct of the pancreas in which PEX3 was very low abundant the peroxisome-bound PEX19 form was present in relatively high amounts. On the other hand, enterocytes, which display high amounts of PEX3 contain only very little cytosolic PEX19. A similar discrepancy was also observed between the abundance of PEX3 and PEX19 in comparison to the one of PEX14. In this study we showed that while the protein abundance of PEX3 and PEX19 greatly varied, the abundance of PEX14 was comparable in nearly all the analysed organs. The differences in the cell type-specific level of peroxisomal biogenesis proteins hint to distinct modulation and fine-tuning of the processes underlying early peroxisome biogenesis and peroxisomal homeostasis. We hypothesize that these adjustments are made in dependence to the differentiation level, metabolism and nutritional and oxidative status of the individual cell types.

**Table 1:** Summary of intensity and particularities of the PEX3 and PEX19 immunofluorescence staining.

Organ	Cell type	PEX3	Staining particularities	PEX19	Staining particularities
**Kidney**	Mesangial cells	(-)po	-	(-/+)cyt; (-)po	-
	Podocytes	(-/+)po	-	(+)cyt; (?)po	-
	Epithelial cells of:				
	- proximal tubule	(++++)po	-	(+++)cyt; (++)po	-
	- intermediate tubule	(-/+)po	sporadic po	(++)cyt; (+)po	-
	- distal tubule	(+++)po	-	(++)cyt; (++)po	More po staining compared to proximal tubule
	- collecting duct	(++)po	-	(++)cyt; (+)po	-
	Endothelial cells	(+)po	-	(-)	-
**Testis**	Leydig cells	(++++)po	-	(+++)cyt; (-)po	-
	Peritubular cells	(-)	-	(-)	-
	Sertoli cells	(++++)po	PEX3 stronger than PEX19	(++)cyt; (?)po	-
	Spermatogonia	(++++)po	PEX3 stronger than PEX19	(+)cyt; (+++)po	-
	Spermatozytes	(++++)po	PEX3 stronger than PEX19	(+)cyt; (++)po	-
	Spermatids	(-)	-	(?)cyt; (-)po	-
**Liver**	Hepatocytes	(+)po	large po clusters	(+)cyt; (++)po	marked around the nucleus
	Epithelial cells of the bile duct	(-/+)po	-	(+++)?	PEX19-labelled clusters
	Endothelial cells	(+)po	-	(-)	-
**Pancreas**	Epithelial cells of the acini	(++)po	cyt autofluorescence; sporadic po	(+++)cyt; (+++)po	sporadic po
	α-cells	(-/+)po	sporadic po	(++)cyt; (++)po	PEX19 stronger than PEX3
	β-cells	(-/+)po	sporadic po	(++)cyt; (++)po	PEX19 stronger than PEX3
	Epithelial cells of the excretory duct	(-/+)po	sporadic po	(+)cyt; (+++)po	PEX19 stronger than PEX3
**Jejunum**	Enterocytes	(+++)po	apically and basally; PEX3 stronger than PEX19	(+)cyt; (-)po	-
	Goblet cells	(+++)po	basally; PEX3 stronger than PEX19	(+)cyt; (-)po	-
	Smooth muscle cells	(-)	-	(-)	-
	Neurons of ganglia	(++)po	-	(-)	-
	Glial cells of ganglia	(-/+)po	-	(-)	-
**Colon**	Enterocytes	(+++)po	apically and basally; PEX3 stronger than PEX19	(+)cyt; (-)po	-
	Goblet cells	(+++)po	basally; PEX3 stronger than PEX19	(+)cyt; (-)po	-
	Smooth muscle cells	(-)	-	(-)	-
	Ganglion cells	(++)po	-	(-)	-
	Glial cells of ganglion	(+)po	-	(-)	-
**Heart**	Cardiomyocytes	(++)po	autofluorescence of myofibrils	(++)cyt; (++++)po	autofluorescence of myofibrils; PEX19 stronger than PEX3
**Skeletal**	Fiber type I	(++)po	autofluorescence of myofibrils	(++)cyt; (+++)po	autofluorescence of myofibrils
**Muscle**	Fiber type IIB	(+)po	autofluorescence of myofibrils	(+)cyt(++)po	autofluorescence of myofibrils
**Lung**	Alveolar type I cell	(-/+)po	sporadic po	(-/+)cyt; (-)po	nuclear staining
	Alveolar type II cell	(+)po	sporadic po	(++)cyt; (?)po	PEX19 stronger than PEX3
	Club cells	(+)po	sporadic po; autofluorescence in cyt	(++++)cyt; (+++)po	PEX19 stronger than PEX3
	Endothelial cells	(+)po	-	(-)	-
**Brain**	Motorneurons	(-/+)po	-	(+++)cyt; (++)po	PEX19 stronger than PEX3
**cortex**	Glia cells	(-/+)po	-	(+)cyt(+)po	PEX19 stronger than PEX3

In this Table we have summarized the staining intensities observed in organ-specific cell-types and indicated the observed staining-specific particularities. (-) no staining; (-/+) staining detectable only after longer exposure times; (+) minimal staining to (++++) very strong staining intensity; (?) unclear staining; po, peroxisome/peroxisomal; cyt, cytosol/cytosolic

### PEX19 displays variable peroxisomal and cytosolic subcellular localisation

The peroxin PEX19 acts as a soluble shuttling receptor: it binds PMPs in the cytosol and recruits them to PEX3 on the peroxisomal membrane [1] and is therefore only transiently associated with peroxisomes. A dual subcellular localisation of PEX19 has been described in earlier publications in yeast [4,5] as well as in mammalian CHO-K1 cells and fibroblasts [51,57], but so far has not been analysed in detail in different organs. Our immunofluorescence analysis of various mouse organs revealed strong variations of the PEX19 protein abundance. Moreover, the amount/ratio of PEX19 localised in peroxisomes and cytosol strongly varied depending on the analysed cell type and organ. In most organs the cytosolic form of PEX19 predominated, corresponding well to previous calculations that indicate that 95% of PEX19 is cytosolic in rat liver [51]. However, in some organs like heart, skeletal muscle or pancreas and spermatogonia in testis, the peroxisome-bound form of PEX19 was particularly high abundant and could easily be detected on top of the cytosolic PEX19 staining. Similar differences were noticed between the MEFs and Hepa 1–6 cells. The dual subcellular localisation of PEX19 is a reflection of its function as a shuttling receptor that targets membrane proteins to the peroxisome by docking to PEX3. However, no studies so far are available on the factors that might influence the ratio of peroxisome-bound to cytosolic PEX19.

Interestingly, the Western blot analysis of the peroxisomal fractions suggests, that the dual subcellular localisation of PEX19 could be connected to a molecular weight shift. Analysis of the peroxisomal fractions revealed a 40 kDa band (liver, testis and brain) and a 50 kDa band (liver, heart, kidney, testis and skeletal muscle) but no band of the expected size of 35 kDa. When we then analysed the immunofluorescence stainings, we found that the parenchyma of the organs displaying a 50 kDa band contained a clear peroxisomal staining that could be visualized next to the cytosolic labelling. High apparent molecular weight for PEX19 has been reported previously in gel filtration experiments in which the protein eluted at around 100 kDa instead of 34 kDa due to its non-globular structure [19,54,68], and in Western blot analyses of total lysates derived from cell-culture and plant seedlings of *Arabidopsis thaliana* where the protein was detected as a dimer [68]. Closer inspection of the Western blot results published by Hadden *et al*. [68] shows that next to the monomeric 30 kDa band and the dimeric 60 kDa band, a number of other products were recognised by the PEX19 antibody in the range between 40 kDa and 55 kDa [68]. *A*. *thaliana* contains two isoforms of PEX19 that are both expressed at the mRNA level, which might contribute to the formation of dimers and heterodimers [68]. Like *A*. *thaliana* also mice contain two differently sized PEX19 isoforms (www.ncbi.nlm.nih.gov): isoform *a* with 299 aa and *b* with 207 aa. The expression of two PEX19 mRNAs is however most likely not the cause for the differently sized PEX19 protein products. Albeit the transient peroxisomal association of PEX19 was described previously for mammals [57] and yeast [4,5], so far this double localisation has never been linked to the formation of dimers or the occurrence of PEX19 isoforms. It would be interesting for future investigations to analyse the nature of these differently sized proteins and to determine their exact subcellular localisation.

Conflicting evidence exists regarding the function of the PEX19 farnesylation at amino acid C347 [4,69]. It was suggested that this modification was required for peroxisomal targeting of PEX19 [57], a result that was disclaimed later by other reports [63,70–72]. Though the farnesylation does not largely influence the molecular weight of a protein *per se*, it might change the lipid binding properties and therefore the behaviour in the SDS-PAGE. Indeed, farnesylation greatly increases the hydrophobicity of proteins changing both subcellular localisation and binding properties [73]. A very recent paper suggested that the farnesylation of PEX19 triggers the insertion of the hydrophobic hairpin-domain containing protein UBDX8 into phospholipid monolayers on the surface of a lipid-droplet and postulated ER subdomains [74]. Ultrastructural analysis will be required to determine the exact nature of these postulated ER subdomains and whether these subdomains are indeed integral parts of the ER-network or subdomains of the peroxisomal membrane compartment [40] containing farnesylated PEX19. We speculate that differences in the subcellular localisation of PEX19 could be due to differences in the rate of farnesylation, e.g. in case of low farnesylation, non-farnesylated cytosolic PEX19 is predominant, whereas at high rates of farnesylation, the farnesylated PEX19, which is localized in the peroxisome is more abundant.

### Possible scenarios for the PEX3- and PEX19-mediated regulation of the peroxisomal homeostasis in different cell types

It is an emerging concept that peroxisomal metabolism and proliferation respond to microenvironmental factors including nutrient supply, oxidative stress and hypoxia and that peroxisomes cross-talk with other organelles for the exchange of metabolic intermediates [31–33,75,76]. In response to these stimuli the abundance of the peroxisomal compartment changed rapidly by either proliferation or degradation [32,77,78], which are regulated at the transcriptional and posttranscriptional level. Transcription factors that regulate peroxisome proliferation are the nuclear receptors of the peroxisome proliferator-activated receptor family (PPARα, β and γ [60,79]. PPARs modulate fatty acid metabolism and are regulated by a variety of short lipid ligands [80,81] by binding to PPAR response elements (PPRE) located in target genes. Experiments showed that they activate the transcription of genes coding for peroxins, peroxisomal transporters as well as β-oxidation and antioxidative enzymes [31,60,82–84]. A PPAR-peroxisome-loop has been suggested for maintaining the PPAR-ligand homeostasis by a feed-back mechanism of peroxisomal β-oxidation genes [60]. PPARs are variably regulated in different tissues and organs and could therefore generate an organ-specific peroxin expression profile by transcriptional control. To investigate the possible regulation of the *Pex3* and *Pex19* genes by PPARs, we have analysed the intergenic region upstream of the transcriptional start using a prediction program developed for the identification of PPREs (www.classicrus.com) and found putative PPARα, β and γ binding sites in the gene of *Pex19*, but none in *Pex3*. This could explain why the abundance of PEX3 and PEX19 is independently regulated although both protein are involved and linked to each other during the early steps of peroxisome biogenesis. However, the functionality of these regulatory elements needs to be experimentally determined in future studies.

Peroxisomal homeostasis is regulated not only by their proliferation, but also by their degradation via pexophagy and other mechanisms [85,86]. Pexophagy is a subtype of autophagy and is induced by different events such as hypoxia through hypoxia-inducible factor 2α-mediated signalling or oxidative stress through ataxia telangiectasia mutated-mediated signalling [76,78,87–89]. Interestingly, both PEX3 and PEX19 were suggested to be involved in pexophagy. It was recently shown that the overexpression of PEX3 induces ubiquitination-dependent NBR1-mediated pexophagy [90–92] and that PEX19 associates with the tuberous sclerosis complex, which is part of the signaling cascade downstream of ATM activating pexophagy in the presence of ROS [88,89]. Next to pexophagy, also peroxisome proliferation is activated by oxidative stress [93–95] and we therefore expected high expression of PEX3 and PEX19 in organs that are exposed to higher ROS levels. However, while the amount of PEX3 and PEX19 in kidney was notable, the expression of these peroxins in the lung or liver, which are also exposed to high levels of oxidative stress [96], was very low. At the moment, it is still unclear how PEX3 and PEX19 contribute to either peroxisome biogenesis or pexophagy in dependence to different ROS levels.

### Conclusions and outlook

In this article we show that the abundance of the closely interacting peroxins PEX3 and PEX19, but also of PEX14, peroxisomal membrane transporters and matrix enzymes, are not regulated in a concerted manner. This indicates that they are differentially controlled by organ- and cell-type-specific signalling networks, which are yet to be identified. Future experiments will clarify how the intracellular levels of PEX3 and PEX19 are differentially modulated in the context of their implication in both peroxisome proliferation and degradation via pexophagy.

## Supporting information

S1 Fig *Pex3* and *Pex19* mRNAs are particularly highly expressed in heart, skeletal muscle and lung. **A and B:** qPCR analyses of *Pex3* (A) and *Pex19* (B) mRNAs using cDNA synthesised from total RNA derived from different mouse organs (as indicated). The three bar graphs display the obtained results normalized (norm) against the following three different reference genes peptidyl prolyl isomerase (*Ppia)*, TATA-box binding protein (Tbp) and ribosomal protein L13 *(Rpl13*). The bar graph with grey columns represents the mean values (Mean) derived from the three black graphs. Values are expressed as fold-change compared to the expression levels obtained for liver, which was set to 1. The error bars represent the standard deviation of three independent experiments.
(TIF)

S2 Fig The amounts of PEX3 and PEX19 and the subcellular localisation of PEX19 vary between the analysed organs. **A and B:** Immunofluorescence analyses of PEX3 (A) and PEX19 (B) in paraffin-embedded sections of mouse organs (as indicated) using the same incubation conditions for all organs. All images were taken with identical camera settings for either the PEX3 or PEX19 staining series to analyse the differences in individual labelling intensities between the organs. Since the labelling intensity for PEX3 was very low in the alveolar region of the lung, the contours of the tissue structure were drawn in grey. Organ sections that were labelled with secondary antibody only were used as negative staining controls (“NC Cy3” for Donkey anti-Rat and “NC 488” for Donkey anti-Rabbit AlexaFluor 488). **Scale bars** = 15 μm.
(TIF)

S3 Fig PEX3 and PEX19 are higher abundant in the bronchiolar than in the alveolar epithelium. **A:** Phase-contrast image of the alveolar region of the lung. **B:** Immunofluorescence analysis of the distribution of PEX3 in the alveolar epithelium shown in A. **C:** Subcellular localisation of PEX3 in the bronchiolar epithelium. **D:** Colocalisation of PEX3 and the PEX14 in an alveolar type II cell. **E:** Phase-contrast image of another region of the alveolar epithelium. **F:** Immunofluorescence analysis of the distribution of PEX19 in the alveolar epithelium shown in E. **G:** Subcellular localisation of PEX19 in the bronchiolar epithelium. **H:** Higher magnification of the alveolar type II cell stained for PEX19 in S2F Fig (square). **I:** Higher magnification of the bronchiolar epithelium stained with PEX19 in S2G Fig (square). **Nuclear stainings:** in Figs B, C, F-I with Hoechst 33342. **Abbreviations:** Da, alveolar duct; Al, alveole; I, alveolar type I cell; II, alveolar type II cell; Br, bronchiole; PhC, phase-contrast. The arrows indicate single labelled peroxisomes. **Scale bars** = 15 μm.
(TIF)

S4 Fig In the brain, PEX19 is highly abundant in the pyramidal neurons of the motorcortex. **A:** Immunofluorescence analysis of the distribution of PEX3 in pyramidal neurons. **B:** 3-fold magnification of a single pyramidal neuron from image A (arrowhead). **C:** Distribution of PEX14 in another pyramidal neuron. **D:** Distribution of PEX19 in apyramidal neurons of the motorcortex. **E:** Higher magnification of pyramidal neurons exhibiting PEX19 staining. **F:** 3-fold magnification of a pyramidal neurons from image E (arrowhead). **Nuclear stainings:** In Figs A-F with Hoechst 33342. **Scale bars** = 15 μm.
(TIF)

S1 Table List of all RT-qPCR primers used in this study. (PDF)

S2 Table List of all primary antibodies used in this study. (PDF)

S3 Table List of all secondary antibodies used for Western blotting. (PDF)

S4 Table List of all secondary antibodies used for immunofluorescence analysis. (PDF)

S5 Table Optimized protocols for the immunofluorescence analysis of mouse tissue using our self-generated antibodies against PEX3 and PEX19. (PDF)

S6 Table Percentage of *Pex19* transcript variant 1 and 2 mRNA level as determined by qPCR. (PDF)

S7 Table Summary of cell-specific staining intensities obtained for PEX14. We have listed the staining intensities observed in organ specific cell-types and indicated noticed staining-specific particularities and the presence of a particularly low number of peroxisomes. **Legend:** (-) no staining; (-/+) staining detectable only after longer exposure times; (+) minimal staining to (++++) very strong staining; (?) unclear staining; po, peroxisome/peroxisomal; cyt, cytosol/cytosolic.
(PDF)
