# Supplementary figures and images for: New insights into the distribution, protein abundance and subcellular localisation of the endogenous peroxisomal biogenesis proteins PEX3 and PEX19 in different organs and cell types of the adult mouse
Source: PLoS One. 2017 Aug 17;12(8):e0183150. doi: 10.1371/journal.pone.0183150 (PMC5560687; doi:10.1371/journal.pone.0183150)

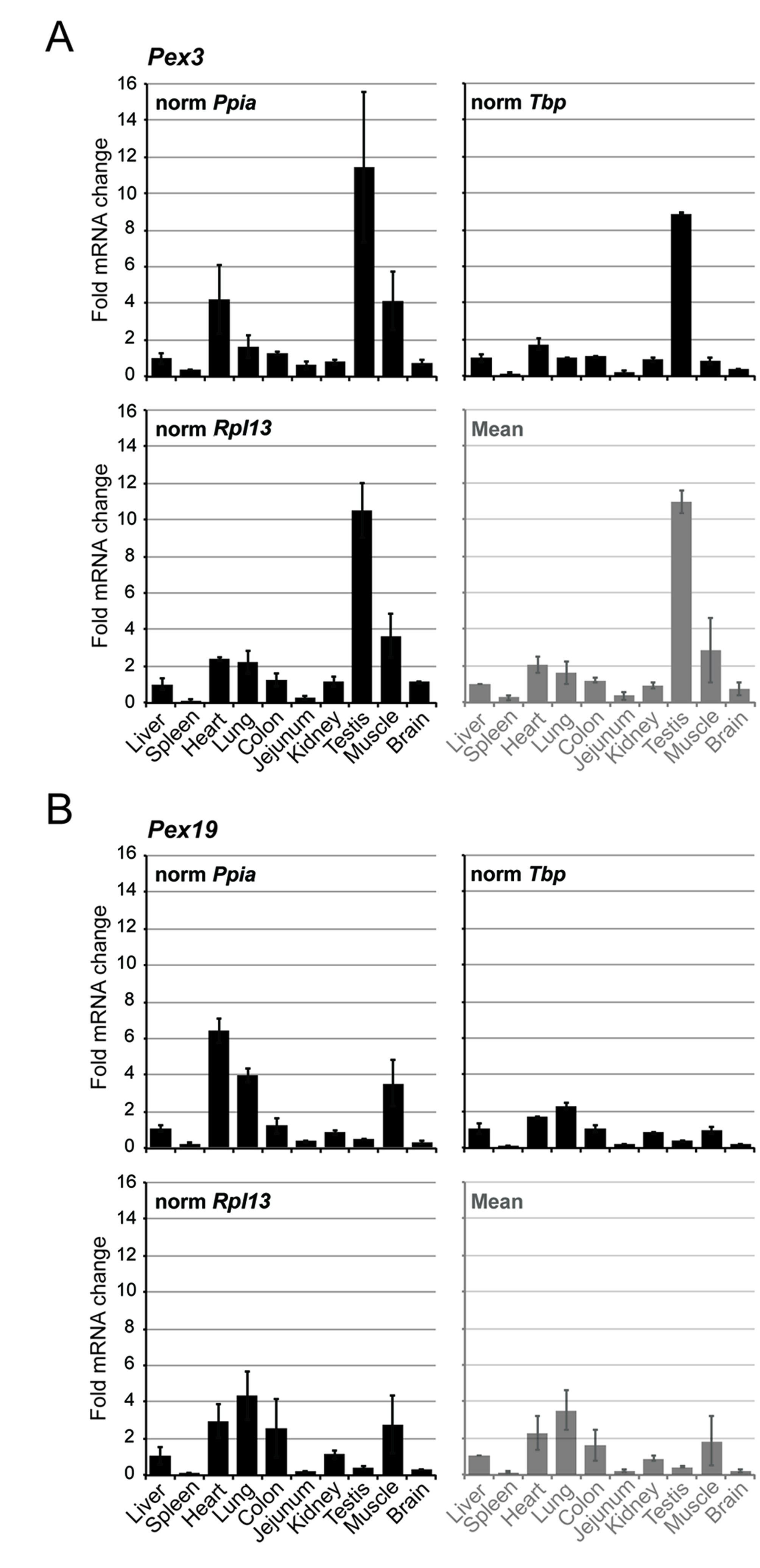

Supplement: S1 Fig — A and B: qPCR analyses of Pex3 (A) and Pex19 (B) mRNAs using cDNA synthesised from total RNA derived from different mouse organs (as indicated). The three bar graphs display the obtained results normalized (norm) against the following three different reference genes peptidyl prolyl isomerase (Ppia), TATA-box binding protein (Tbp) and ribosomal protein L13 (Rpl13). The bar graph with grey columns represents the mean values (Mean) derived from the three black graphs. Values are expressed as fold-change compared to the expression levels obtained for liver, which was set to 1. The error bars represent the standard deviation of three independent experiments. (TIF) [file pone.0183150.s001.tif]

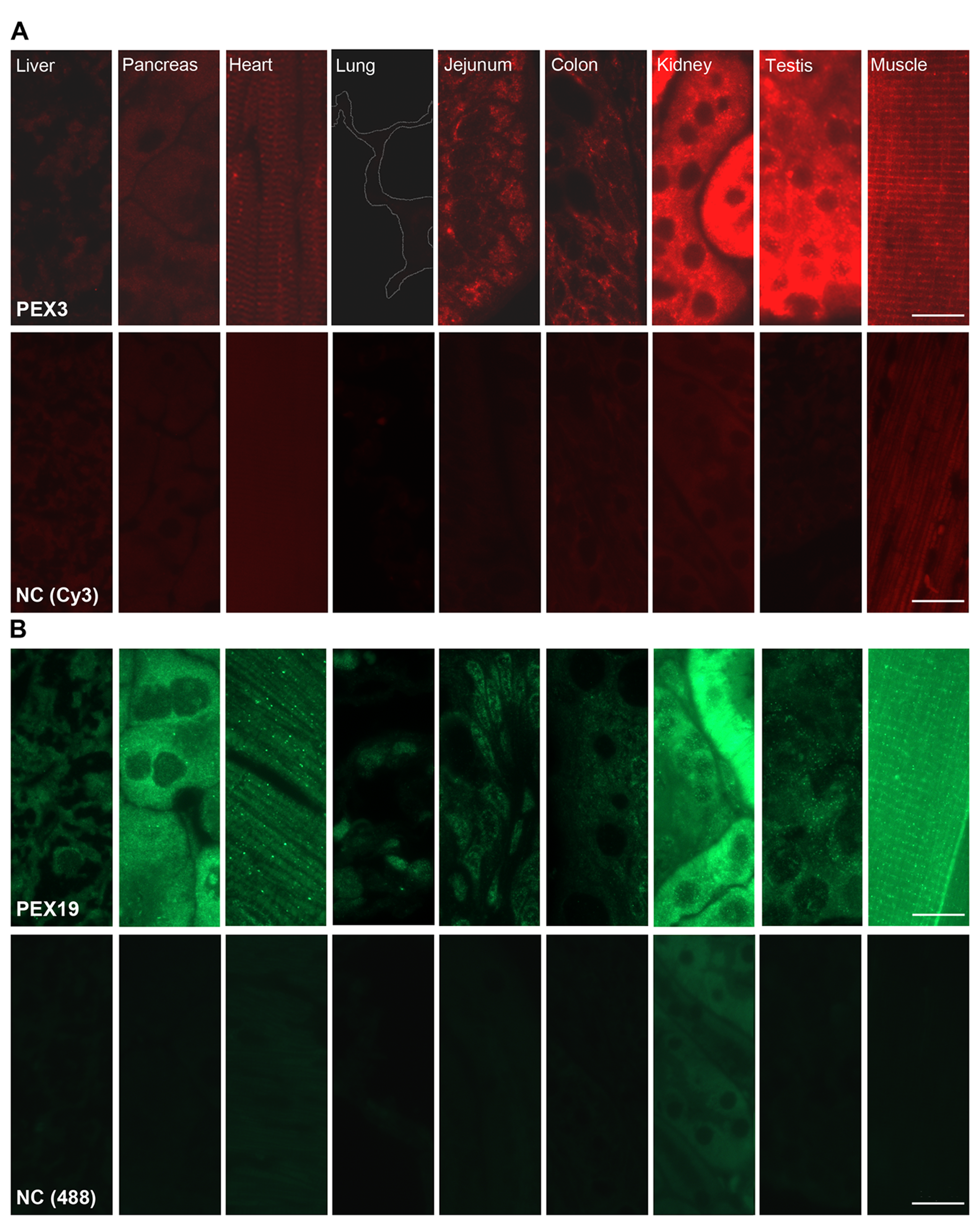

Supplement: S2 Fig — A and B: Immunofluorescence analyses of PEX3 (A) and PEX19 (B) in paraffin-embedded sections of mouse organs (as indicated) using the same incubation conditions for all organs. All images were taken with identical camera settings for either the PEX3 or PEX19 staining series to analyse the differences in individual labelling intensities between the organs. Since the labelling intensity for PEX3 was very low in the alveolar region of the lung, the contours of the tissue structure were drawn in grey. Organ sections that were labelled with secondary antibody only were used as negative staining controls (“NC Cy3” for Donkey anti-Rat and “NC 488” for Donkey anti-Rabbit AlexaFluor 488). Scale bars = 15 μm. (TIF) [file pone.0183150.s002.tif]

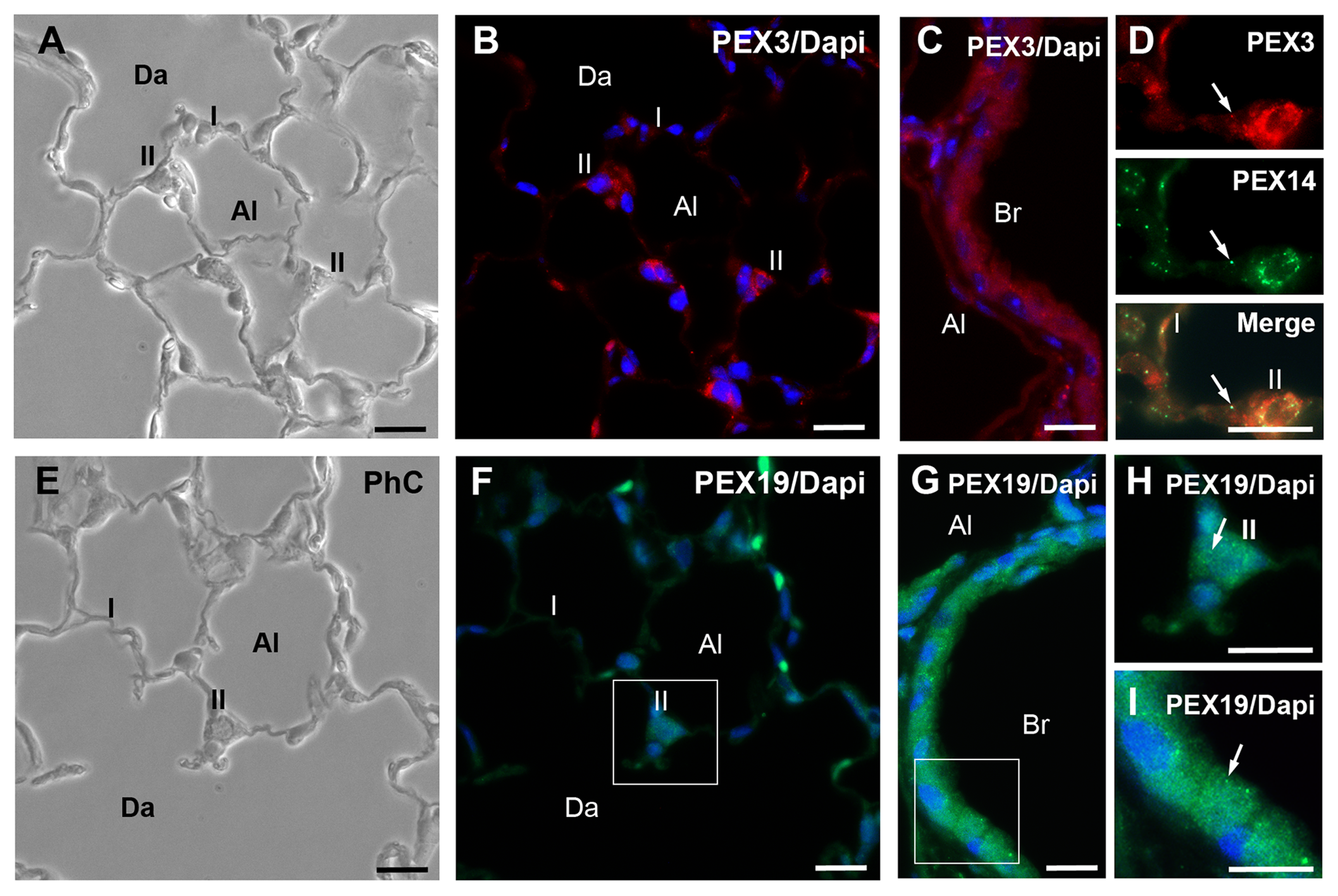

Supplement: S3 Fig — A: Phase-contrast image of the alveolar region of the lung. B: Immunofluorescence analysis of the distribution of PEX3 in the alveolar epithelium shown in A. C: Subcellular localisation of PEX3 in the bronchiolar epithelium. D: Colocalisation of PEX3 and the PEX14 in an alveolar type II cell. E: Phase-contrast image of another region of the alveolar epithelium. F: Immunofluorescence analysis of the distribution of PEX19 in the alveolar epithelium shown in E. G: Subcellular localisation of PEX19 in the bronchiolar epithelium. H: Higher magnification of the alveolar type II cell stained for PEX19 in S2F Fig (square). I: Higher magnification of the bronchiolar epithelium stained with PEX19 in S2G Fig (square). Nuclear stainings: in Figs B, C, F-I with Hoechst 33342. Abbreviations: Da, alveolar duct; Al, alveole; I, alveolar type I cell; II, alveolar type II cell; Br, bronchiole; PhC, phase-contrast. The arrows indicate single labelled peroxisomes. Scale bars = 15 μm. (TIF) [file pone.0183150.s003.tif]

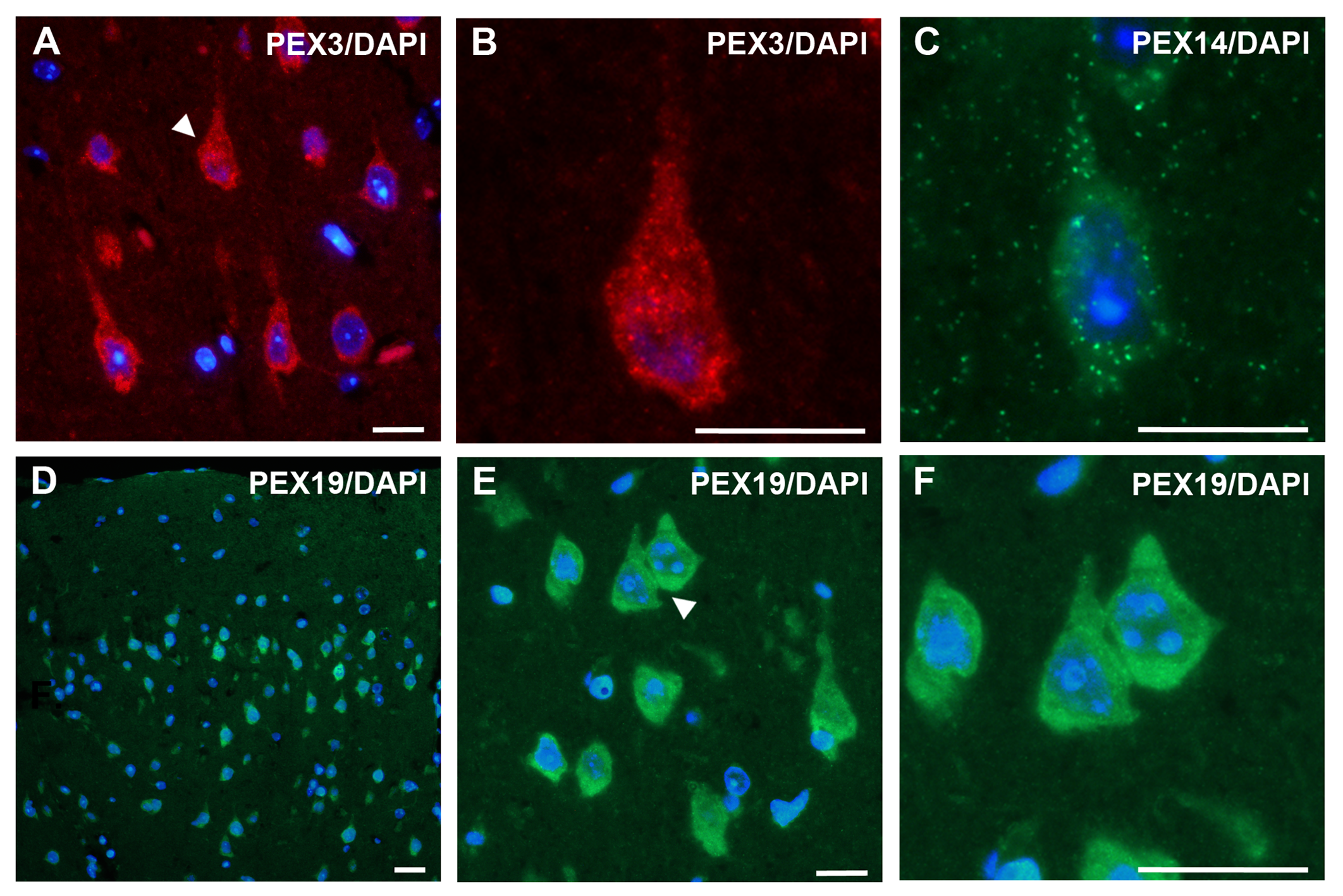

Supplement: S4 Fig — A: Immunofluorescence analysis of the distribution of PEX3 in pyramidal neurons. B: 3-fold magnification of a single pyramidal neuron from image A (arrowhead). C: Distribution of PEX14 in another pyramidal neuron. D: Distribution of PEX19 in apyramidal neurons of the motorcortex. E: Higher magnification of pyramidal neurons exhibiting PEX19 staining. F: 3-fold magnification of a pyramidal neurons from image E (arrowhead). Nuclear stainings: In Figs A-F with Hoechst 33342. Scale bars = 15 μm. (TIF) [file pone.0183150.s004.tif]
