# Supplementary material for: New insights into the distribution, protein abundance and subcellular localisation of the endogenous peroxisomal biogenesis proteins PEX3 and PEX19 in different organs and cell types of the adult mouse
Source: PLoS One. 2017 Aug 17;12(8):e0183150. doi: 10.1371/journal.pone.0183150 (PMC5560687; doi:10.1371/journal.pone.0183150)
Supplement: S1 Table — (PDF) [file pone.0183150.s005.pdf]

S1 Table

Gene	NCBI Reference	3´- 5´ Sequence	Size (bp)	Location	Efficiency
RNA polymerase II<br>*Pol2ra*	NM_001291068.1	GAGTCCAGAACGAGTGCATGA<br>ACAGGCAACACTGTGACAATC	117	1058-1078<br>1174-1154	1.99
Tata-Box binding Protein<br>*Tbp*	NM_013684.3	TAACCCAGAACATTGAAGAC<br>GCCAAGCCCTGAGCATAA	128	164-183<br>291-274	1.99<br>spleen: 1.94
Ribosomal Protein L13A<br>*Rpl13a*	NM_009438.5	AGCCTACCAGAAAGTTTGCCTTAC<br>GCTTCTTCTTCCGATAGTGCATC	129	420-438<br>548-526	1.99
Peptidylprolyl isomerase A<br>*Ppia*	NM_008907.1	AATGCTGGACCAAACACAAA<br>TTCCACAATGTTCATGCCTT	117	345-364<br>461-442	1.99<br>muscle: 1.98
Peroxin 3<br>*Pex3*	NM_001164195.1	CTGAGCAGGACCTACAGCATG<br>CCATCATCAGCAGATCCTGCAC	150	1146-1166<br>1295-1274	1.99<br>spleen: 1.96
Peroxin 19 (isoforms 1 and 2)<br>*Pex19*	NM_001159525.1<br>NM_023041.3	CAGCAGCACAGCGTCATGGTCA<br>GTTGAGGCCAGGAGGCATCT	174	682-703<br>855-836	1.99
Peroxin 19 (isoform 1 specific)<br>*Pex19*	NM_023041.3	CACCAGTAAGATGGCGGCTGCTGA<br>TTGGGAGGCGAAGAGAGCAT	208	18-38<br>225-206	1.99
