# Supplementary material for: New insights into the distribution, protein abundance and subcellular localisation of the endogenous peroxisomal biogenesis proteins PEX3 and PEX19 in different organs and cell types of the adult mouse
Source: PLoS One. 2017 Aug 17;12(8):e0183150. doi: 10.1371/journal.pone.0183150 (PMC5560687; doi:10.1371/journal.pone.0183150)
Supplement: S2 Table — (PDF) [file pone.0183150.s006.pdf]

S2 Table

Primary antibody target	Host species	Best dilution Western blot	Best dilution immunofluorescence
Mouse PEX19	Rabbit	1:20,000	1:10,000 (cells and organs except brain) 1:5,000 (brain)
Mouse PEX3	Rat	1:10,000	1:200 (heart, colon, lung, skeletal muscle and brain) 1:500 (kidney, liver, jejunum, pancreas and cells) 1:1,000 (testis)
Human PMP70<br>Gift from Steve Gould (Braiterman LT et al. Hum Mol Genet. 1998;7: 239-47)	Sheep	1:2,000	1:500
Mouse PEX14<br>Gift from Denis I. Crane (Grant P et al. Histochem Cell Biol. 2013;140: 423–442)	Rabbit	1:10,000	1:5,000 (cells and organs)
Mouse catalase<br>Gift from Denis I. Crane (Grant P et al. Histochem Cell Biol. 2013;140: 423–442)	Rabbit	1:2,000	1:2,000
His-Tag<br>Cell signalling	Rabbit	1:1,000	-
SOD2<br>Abcam	Rabbit	1:7,000	-
GAPDH<br>HyTest Ltd., Intelligate, Turku, Finland	Mouse	1:20,000	-
