# Supplementary material for: New insights into the distribution, protein abundance and subcellular localisation of the endogenous peroxisomal biogenesis proteins PEX3 and PEX19 in different organs and cell types of the adult mouse
Source: PLoS One. 2017 Aug 17;12(8):e0183150. doi: 10.1371/journal.pone.0183150 (PMC5560687; doi:10.1371/journal.pone.0183150)
Supplement: S3 Table — (PDF) [file pone.0183150.s007.pdf]

S3 Table

Secondary antibody	Dilution	Detection system
Goat anti-rabbit IgG (Sigma)	1:20,000	ECL (peroxidase) (Biorad)
Goat anti-rat IgG (Sigma)	1:4,000	Immun-Star™-AP (Biorad)
Goat anti-mouse IgG (Sigma)	1:20,000	ECL (peroxidase) (Biorad)
