# Supplementary material for: New insights into the distribution, protein abundance and subcellular localisation of the endogenous peroxisomal biogenesis proteins PEX3 and PEX19 in different organs and cell types of the adult mouse
Source: PLoS One. 2017 Aug 17;12(8):e0183150. doi: 10.1371/journal.pone.0183150 (PMC5560687; doi:10.1371/journal.pone.0183150)
Supplement: S4 Table — (PDF) [file pone.0183150.s008.pdf]

S4 Table

Species	Dilution	Fluorochrome
Donkey anti-rabbit (Molecular probes)	1:300	AlexaFluor® 488
Donkey anti-rat (Dianova)	1:1,200	Cy3
Donkey anti-sheep (Molecular probes)	1:500	AlexaFluor® 594
