# Supplementary material for: New insights into the distribution, protein abundance and subcellular localisation of the endogenous peroxisomal biogenesis proteins PEX3 and PEX19 in different organs and cell types of the adult mouse
Source: PLoS One. 2017 Aug 17;12(8):e0183150. doi: 10.1371/journal.pone.0183150 (PMC5560687; doi:10.1371/journal.pone.0183150)
Supplement: S5 Table — (PDF) [file pone.0183150.s009.pdf]

S5 Table

Organs	Proteolysis (0.01% Trypsin)	Citrate Buffer microwaving	Antibody dilution PEX3	Antibody dilution PEX19
Liver	10 min	3 x 5 min	1:500	1:10,000
Pancreas	8 min	3 x 5 min	1:500	1:10,000
Heart	12 min	3 x 5 min	1:200	1:10,000
Lung	10 min	3 x 5 min	1:200	1:10,000
Jejunum	10 min	3 x 5 min	1:500	1:10,000
Colon	10 min	3 x 5 min	1:200	1:10,000
Kidney	10 min	3 x 5 min	1:500	1:10,000
Testis	10 min	3 x 5 min	1:1,000	1:10,000
Skeletal muscle	12 min	3 x 5 min	1:200	1:10,000
Brain	16 min	3 x 5 min	1:200	1:5,000
