# Supplementary material for: New insights into the distribution, protein abundance and subcellular localisation of the endogenous peroxisomal biogenesis proteins PEX3 and PEX19 in different organs and cell types of the adult mouse
Source: PLoS One. 2017 Aug 17;12(8):e0183150. doi: 10.1371/journal.pone.0183150 (PMC5560687; doi:10.1371/journal.pone.0183150)
Supplement: S6 Table — (PDF) [file pone.0183150.s010.pdf]

S6 Table

Organ samples	*Pex19* transcript variant 1 (%)	*Pex19* transcript variant 2 (%)
Liver	50	50
Spleen	100	0
Heart	75	25
Lung	100	0
Colon	85	15
Jejunum	80	20
Kidney	75	25
Testis	100	0
Skeletal muscle	100	0
Brain	65	35
