# Supplementary material for: New insights into the distribution, protein abundance and subcellular localisation of the endogenous peroxisomal biogenesis proteins PEX3 and PEX19 in different organs and cell types of the adult mouse
Source: PLoS One. 2017 Aug 17;12(8):e0183150. doi: 10.1371/journal.pone.0183150 (PMC5560687; doi:10.1371/journal.pone.0183150)
Supplement: S7 Table — We have listed the staining intensities observed in organ specific cell-types and indicated noticed staining-specific particularities and the presence of a particularly low number of peroxisomes. Legend: (-) no staining; (-/+) staining detectable only after longer exposure times; (+) minimal staining to (++++) very strong staining; (?) unclear staining; po, peroxisome/peroxisomal; cyt, cytosol/cytosolic. (PDF) [file pone.0183150.s011.pdf]

**S7 Table**

| Organ | Cell type | PEX14 | Staining particularities |
|---|---|---|---|
| Kidney | Mesangial cells | (-/+)po | - |
| | Podocytes | (-/+)po | sporadic po |
| | Epithelial cells of: | | |
| | - proximal tubule | (++++)po | - |
| | - intermediate tubule | (-/+)po | sporadic po |
| | - distal tubule | (+++)po | - |
| | - collecting duct | (++)po | - |
| | Endothelial cells | (+)po | - |
| Testis | Leydig cells | (+)po | cyt background |
| | Peritubular cells | (-/+)po | - |
| | Sertoli cells | (++++)po | - |
| | Spermatogonia | (+++)po | - |
| | Spermatocytes | (+++)po | - |
| | Round spermatids | (++++)po | peroxisomal clusters in late stages |
| Liver | Hepatocytes | (++++)po | - |
| | Epithelial cells of the bile duct | (-/+)po | sporadic po |
| | Endothelial cells | (+)po | - |
| Pancreas | Epithelial cells of the acini | (+++)po | cyt autofluorescence; sporadic po |
| | $\alpha$-cells | (++++)po | - |
| | $\beta$-cells | (++++)po | - |
| | Epithelial cells of the excretory duct | (+++)po | sporadic po |
| Jejunum | Enterocytes | (++++)po | apically and basally |
| | Goblet cells | (++++)po | basally |
| | Smooth muscle cells | (-/+)po | - |
| | Neurons of ganglia | (+++)po | - |
| | Glial cells of ganglia | (-/+)po | - |
| Colon | Enterocytes | (+++)po | apically and basally |
| | Goblet cells | (+++)po | basally |
| | Smooth muscle cells | (-/+)po | - |
| | Ganglion cells | (+++)po | - |
| | Glial cells of ganglion | (-/+)po | - |

| | | | |
|---|---|---|---|
| **Heart** | Cardiomyocytes | (++++)po | - |
| **Skeletal muscle** | Fiber type I | (++++)po | - |
| | Fiber type IIB | (++++)po | - |
| **Lung** | Alveolar type I cell | (+++)po | sporadic po |
| | Alveolar type II cell | (++++)po | sporadic po |
| | Club cells | (+++)po | sporadic po; autofluorescence in cyt |
| | Endothelial cells | (+)po | - |
| **Brain cortex** | Motorneuron | (+++)po | - |
| | Glia cells | (+++)po | - |
